# Bifacial Passivation of Organic Hole Transport Interlayer for NiO*_x_*‐Based p‐i‐n Perovskite Solar Cells

**DOI:** 10.1002/advs.201802163

**Published:** 2019-01-29

**Authors:** Zijia Li, Bong Hyun Jo, Su Jin Hwang, Tae Hak Kim, Sivaraman Somasundaram, Eswaran Kamaraj, Jiwon Bang, Tae Kyu Ahn, Sanghyuk Park, Hui Joon Park

**Affiliations:** ^1^ Department of Energy Science Sungkyunkwan University Suwon 16419 Republic of Korea; ^2^ Department of Chemistry Kongju National University Kongju 32588 Republic of Korea; ^3^ Department of Energy Systems Research Ajou University Suwon 16499 Republic of Korea; ^4^ Nano Convergence Materials Center Korea Institute of Ceramic Engineering and Technology Jinju 52851 Republic of Korea; ^5^ Department of Electrical and Computer Engineering Ajou University Suwon 16499 Republic of Korea

**Keywords:** defect passivation, hole transport materials, perovskite solar cells, UV durability

## Abstract

Methoxy‐functionalized triphenylamine‐imidazole derivatives that can simultaneously work as hole transport materials (HTMs) and interface‐modifiers are designed for high‐performance and stable perovskite solar cells (PSCs). Satisfying the fundamental electrical and optical properties as HTMs of p‐i‐n planar PSCs, their energy levels can be further tuned by the number of methoxy units for better alignment with those of perovskite, leading to efficient hole extraction. Moreover, when they are introduced between perovskite photoabsorber and low‐temperature solution‐processed NiO*_x_* interlayer, widely featured as an inorganic HTM but known to be vulnerable to interfacial defect generation and poor contact formation with perovskite, nitrogen and oxygen atoms in those organic molecules are found to work as Lewis bases that can passivate undercoordinated ion‐induced defects in the perovskite and NiO*_x_* layers inducing carrier recombination, and the improved interfaces are also beneficial to enhance the crystallinity of perovskite. The formation of Lewis adducts is directly observed by IR, Raman, and X‐ray photoelectron spectroscopy, and improved charge extraction and reduced recombination kinetics are confirmed by time‐resolved photoluminescence and transient photovoltage experiments. Moreover, UV‐blocking ability of the organic HTMs, the ameliorated interfacial property, and the improved crystallinity of perovskite significantly enhance the stability of PSCs under constant UV illumination in air without encapsulation.

## 1 Introduction

Organic–inorganic hybrid perovskite solar cell (PSC) has been receiving great attention in optoelectronic fields due to its fascinating optical and electrical features such as high absorption coefficient, long diffusion length and lifetime, and bipolar carrier transport.^1^, ^2^ Moreover, its cost‐effective low‐temperature solution processability makes it a good candidate for next‐generation solar cell.^3^ Along with the development of fabrication techniques, the advances in perovskite and charge transport materials including interfacial layers, the maximum power conversion efficiencies (PCEs) of PSCs have exceeded 22% now since their first application to solar cells.^4^, ^5^

The PSC device architectures can be classified into two types, a conventional n‐i‐p structure (planar and mesoscopic) and an inverted p‐i‐n structure.^6^, ^7^, ^8^ The record efficiency was from the n‐i‐p PSCs employing titanium oxide (TiO_2_) mesoporous (mp) structure as an electron‐transport layer (ETL), but the inevitable high‐temperature (over 400 °C) sintering process to create a conductive phase has hindered its flexible and large‐scale applications for future commercialization.^4^, ^5^, ^9^, ^10^ Furthermore, the planar n‐i‐p type PSCs without mp structure usually suffer from the severe hysteretic current density (*J*)–voltage (*V*) characteristics depending on the scan direction and rate.^11^, ^12^, ^13^ Those hysteretic behaviors are shown to be repressed in the p‐i‐n structure PSCs, because a little shorter hole diffusion length than the electron can be compensated in this architecture, improving hole extraction,^14^, ^15^ and fullerene derivative, often utilized as an ETL, is known to passivate defect sites of perovskite.^16^ In addition to this merit, the low‐temperature process feasibility has aroused extensive researches on this device architecture for low‐cost flexible PSCs.^17^

Employing suitable hole‐transporting layers (HTLs), which can reduce the energy losses of photogenerated carriers and minimize the interfacial charge recombination, is a prerequisite to achieve high‐performance p‐i‐n PSCs. Moreover, optimal selection of HTLs can enhance the device stability against constant full‐spectrum solar irradiation in ambient air by restraining the ion migration^18^, ^19^, ^20^ and improving the crystallinity of the active layer.^21^, ^22^, ^23^ The conventional organic HTLs such as poly(3,4‐ethylenedioxythiophene):poly(styrene sulfonate) (PEDOT:PSS),^24^, ^25^ spiro‐OMeTAD,^26^, ^27^, ^28^ and poly(triarylamine) (PTAA)^29^, ^30^ may be able to impede the commercialization of PSCs due to their high‐price and dopant‐induced poor stability. By comparison, the inorganic HTLs such as CuGaO_2_,^31^ NiO*_x_*,^32^, ^33^, ^34^, ^35^ CuCrO_2_,^36^, ^37^ CuO*_x_*,^38^, ^39^ CuSbS_2_,^40^, ^41^, ^42^ and CuSCN^43^ exhibit low cost, superior stability and high carrier mobility without corrosion to the photoactive layer. Especially, low‐temperature‐processed NiO*_x_* is one of the most investigated due to its large bandgap with high transmittance in visible range, deep valence band edge, and intrinsic p‐type conductivity.^32^, ^34^ However, the low‐temperature solution processes usually increase the defects by deteriorating its crystallinity, which results in a series of deficiencies like reduced hole extraction and increased carrier recombination.^44^, ^45^ The interstitial vacancies and defects in NiO*_x_* thin films incur hole accumulation near the perovskite interface with the trap‐assisted nonradiative recombination losses, consequently decreasing the device performances. In addition, those defects induce rough contact with the perovskite layer, which degrades the crystallinity and morphology of perovskite layer with lower light absorption.^14^, ^15^, ^46^ Therefore, controlling the interface between NiO*_x_* HTL and perovskite photoactive layer can be an efficient way to improve the overall performance and stability of the PSCs having the low‐temperature‐processed NiO*_x_*.^14^, ^47^

In this work, a series of triphenylamine‐imidazole derivatives with different number of methoxy group, which can work as HTLs and surface modifiers simultaneously, are designed and applied to the interface between NiO*_x_* and perovskite in p‐i‐n type PSCs. From their electrical and optical properties, it is confirmed that those molecules are proper to the HTL in p‐i‐n PSCs (e.g., high hole mobility, proper energy level, and visible transparency) and the addition of methoxy moieties to backbone deepens their highest occupied molecular orbital (HOMO) levels that can improve built‐in potential of devices for better hole extraction. Moreover, N and O atoms in the HTL molecules are shown to form Lewis adducts with undercoordinated Ni and Pb ions in NiO*_x_* and perovskite, bifacially passivating those defect sites and improving the crystallinity of perovskite. Consequently, the PSCs having those organic HTLs even without dopant represent improved hole extraction and reduced charge recombination behaviors, and these enhanced properties are more discernible by increasing the number of methoxy units in the triphenylamine‐imidazole conjugation scaffold. Moreover, the UV‐blocking ability of the organic HTLs, the ameliorated interfacial property, and the improved crystalinity of perovskite significantly enhance the stability of PSCs under constant UV illumination in air without encapsulation.

## 2 Results and Discussion

### 2.1 Material Design

**Figure 1:**
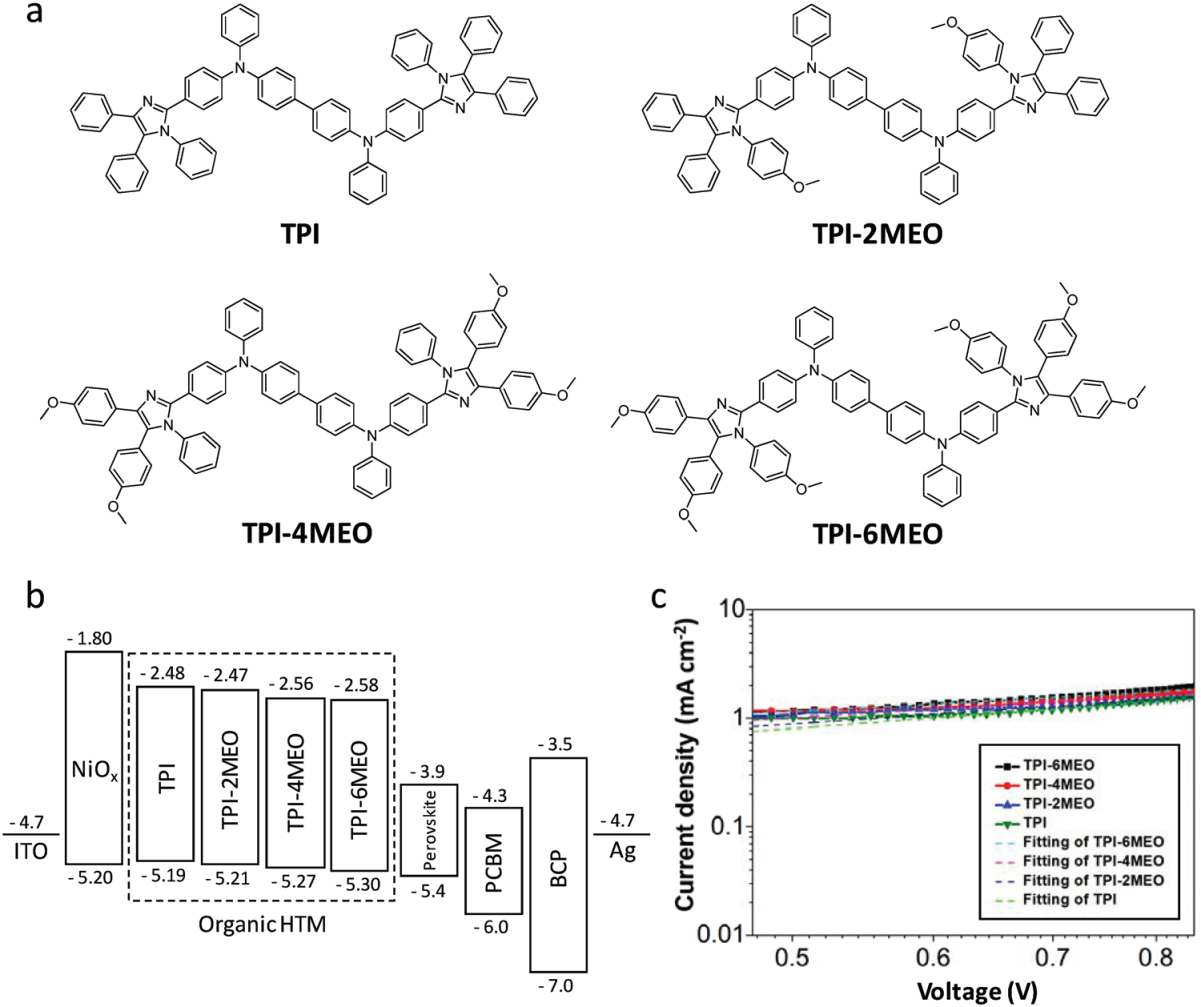
a) Detailed chemical structures of the designed methoxy‐functionalized triphenylamine‐imidazole derivatives for dopant‐free hole‐transporting materials. b) Energy level of each layer of the perovskite solar cell. c) Measured log*J*–log*V* plots of hole‐only devices under dark condition for the estimation of hole mobility of HTM. The calculated hole mobility values of TPI, TPI‐2MEO, TPI‐4MEO, and TPI‐6MEO from the SCLC method are 2.1 × 10^−5^, 2.5 × 10^−5^, 3.3 × 10^−5^, and 4.8 × 10^−5^ cm^2^ V^−1^ s^−1^, respectively.

**Figure** **1**a shows the synthesized organic molecules sharing the same conjugated backbone with the different number of methoxy unit (TPI, TPI‐2MEO, TPI‐4MEO, and TPI‐6MEO having 0, 2, 4, and 6 methoxy units, respectively). The molecules could be synthesized by the straightforward two reaction steps from commercially available simple reagents with high reaction yield as explained in Figure S1 and Materials Synthesis section (Supporting Information). Materials characterization data such as ^1^H NMR (Figures S2, S4, S7, S10, and S13, Supporting Information), ^13^C NMR (Figures S3, S5, S8, S11, and S14, Supporting Information), and electrospray ionization‐mass spectrometry (ESI‐MS) (Figures S6, S9, S12, and S15, Supporting Information) are given in the Supporting Information. Particularly, their backbones were designed to be acceptor–donor–acceptor (A‐D‐A) type conjugated molecules. The electron‐rich triphenylamine dimer structure was utilized as the core of the materials due to its efficient hole transport mobility, and ambipolar imidazole derivatives were added as the acceptor due to their relative electron deficiency. In this design, the strong intermolecular interaction between backbones increases the probability of close molecule–molecule contacts facilitating charge carrier transport, and moreover, their optical and electrical properties can be easily tunable to be a HTM of PSCs.

Figure 1b represents that HOMO energy levels of designed molecules are well‐aligned with the valence band edge of perovskite (−5.4 eV) for efficient hole extraction and their lowest unoccupied molecular orbital (LUMO) energy levels are shallow enough to block the electron transfer from the perovskite. The HOMO energy levels of molecules were obtained by cyclic voltammetry (CV) results in Figure S16 (Supporting Information), and the LUMO energy levels were estimated by their absorption onset in Figure S17 (Supporting Information) and CV results. By introducing electron‐donating methoxy groups to the phenylimidazole fragments, which are electron‐accepting part in dumbbell‐shaped A‐D‐A molecules, it is confirmed that further fine‐tuning of the HOMO level is possible. The addition of methoxy groups to backbone deepens their HOMO levels (Figure 1b), advantageous to improve built‐in potential of devices enhancing their hole extraction property.^7^ Along with the variation of HOMO levels, methoxy groups are expected to function as Lewis base that can passivate defect sites of perovskite at the interface,^48^, ^49^ and this will be discussed with Raman, X‐ray photoelectron spectroscopy (XPS), and transient experiment results later again. Moreover, different from the conventional D‐A structured HTMs, applied to n‐i‐p PSCs having the absorption in the visible region, the bandgaps of molecules were adjusted for absorbing the light below 420 nm wavelength not to decrease the amount of incident photon transmitted into the perovskite photoabsorber in p‐i‐n configuration PSCs, and only the absorption around 280 nm wavelength region increased with the addition of methoxy unit, as shown in the absorbance spectra (Figure S17, Supporting Information). Meanwhile, the hole mobility values of the organic HTL materials were estimated by a space‐charge limited current method using the Mott–Gurney law: *J* = 9ε_o_ε_r_ *µV* ^2^/8*L* ^3^, where ε_o_ε_r_ is the permittivity of the component, *µ* is the carrier mobility, and *L* is the thickness,^50^ as shown in Figure 1c, and their values were comparable to that of the conventional organic HTL material such as spiro‐OMeTAD in the literature.^51^

### 2.2 PSC Device Performances

**Figure 2:**
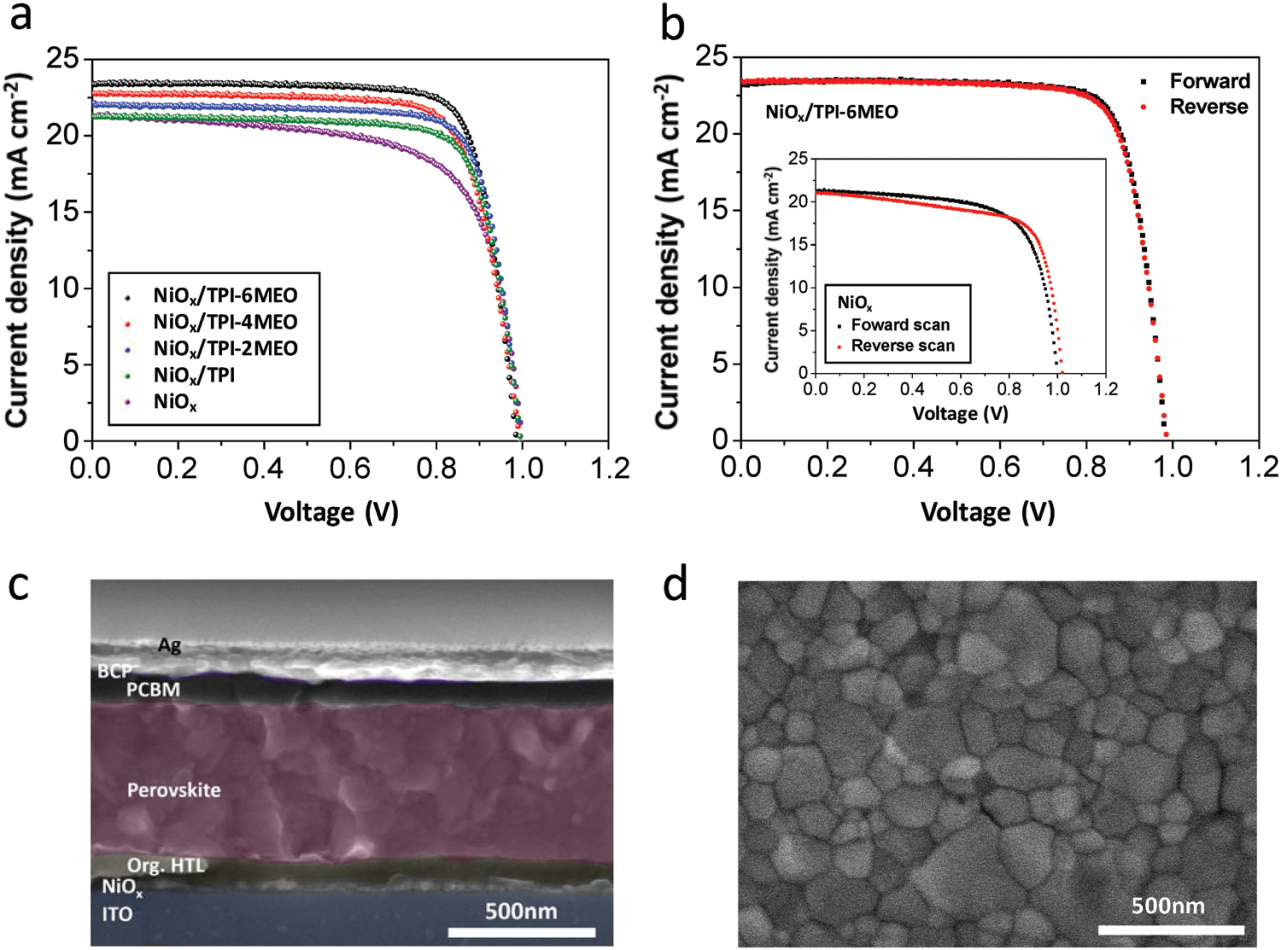
a) *J*–*V* curves of PSCs without and with organic HTLs (TPI, TPI‐2MEO, TPI‐4MEO, and TPI‐6MEO) on NiO*_x_* layer. Device configuration is ITO/NiO*_x_*/organic HTL/CH_3_NH_3_PbI_3_/PCBM/BCP/Ag. All data were measured at AM 1.5 G (100 mW cm^−2^ intensity). b) *J*–*V* curves of PSC built on TPI‐6MEO‐cast NiO*_x_* layer, scanned in forward and reverse directions. Inset figure is *J*–*V* curves of PSC built on bare NiO*_x_* layer, scanned in forward and reverse directions. c) The cross‐sectional SEM image of device (TPI‐6MEO‐applied PSC). d) The top‐view SEM image of perovskite prepared on organic HTL (TPI‐6MEO).

**Figure** **2**a,b and **Table** **1** show *J*–*V* characteristics of p‐i‐n PSC devices with and without organic HTL having the following configuration: ITO/NiO*_x_*/organic HTL/perovskite/PCBM/bathocuproine (BCP)/Ag. The scanning electron microscopy (SEM) images of the cross section of device and the surface of perovskite prepared on an organic HTL are shown in Figure 2c,d. All devices with the organic HTL have improved performances (TPI: 15.92, TPI‐2MEO: 16.56, TPI‐4MEO: 17.59, and TPI‐6MEO: 18.42% PCE), compared to that without the organic HTL (14.57% PCE), and the PSCs with the organic HTL having the highest number of methoxy unit (TPI‐6MEO) represent the best performances, which are mainly ascribed to the enhanced short‐circuit current density (*J*_sc_) and fill factor (FF). Especially, the slightly hysteretic *J*–*V* characteristics, observed from the pristine NiO*_x_*‐based PSCs, are suppressed in the PSCs with the organic HTLs, and *J*–*V* curves of a pristine NiO*_x_*‐based PSC and a TPI‐6MEO‐added PSC depending on the scan direction are in Figure 2b. The stable operation of PSCs with the organic HTLs was further confirmed by the steady‐state output power at the maximum power point (MPP), and, for TPI‐6MEO‐added PSC as a representative, ≈18.3% of PCE was measured from MPP, comparable to that from its *J*–*V* characteristics (Figure S18, Supporting Information). Meanwhile, the external quantum efficiency (EQE) signals of PSCs show similar trend to their *J*–*V* characteristics as shown in Figure S19 (Supporting Information).

**Table 1:** Parameters of the PSCs with various HTMs measured by *J*–*V* characteristics

HTLa)	*J*_sc_ a) [mA cm^−2^]	*V*_oc_ a) [V]	FFa)	PCEa) [%]
Pristine NiO*_x_*	20.52 (21.31)	0.99 (0.99)	0.67 (0.69)	13.61 (14.57)
TPI	20.66 (21.34)	0.98 (0.99)	0.74 (0.75)	14.98 (15.92)
TPI‐2MEO	21.40 (22.01)	0.98 (0.99)	0.74 (0.76)	15.52 (16.56)
TPI‐4MEO	22.58 (22.78)	0.99 (0.99)	0.76 (0.78)	16.99 (17.59)
TPI‐6MEO	23.18 (23.31)	0.97 (0.98)	0.79 (0.81)	17.76 (18.42)

^a)^ Numbers are average values of forward and reverse scan data, measured from at least over 30 devices for each condition (values in parentheses are from the best performing devices).

### 2.3 Defect Passivation of Organic HTM

**Figure 3:**
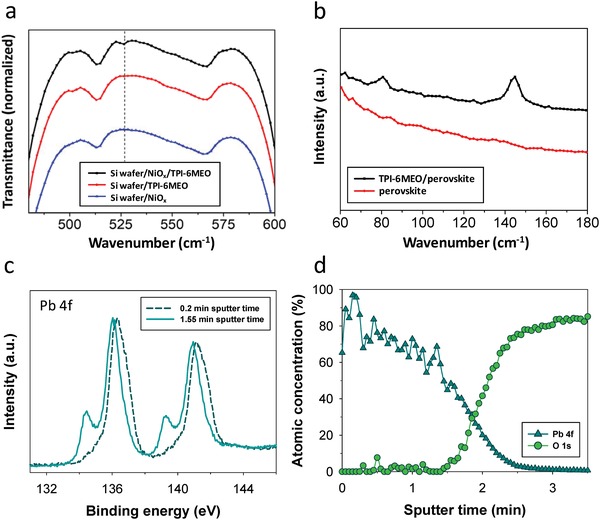
a) IR spectra of TPI‐6MEO‐only layer, NiO*_x_*‐only layer, and TPI‐6MEO‐cast NiO*_x_* layer on Si wafer substrate. b) Raman spectra of perovskite‐only layer and perovskite on TPI‐6MEO layer. c) XPS spectra showing the Pb 4f region of the perovskite on TPI‐6MEO film at 0.2 and 1.55 min sputtering time. d) XPS depth profiles of the perovskite on TPI‐6MEO film.

To clarify the improved performances of PSCs with organic HTLs, the interface between NiO*_x_* and organic HTL was investigated by IR spectroscopy. The oxygen vacancy‐induced Ni^3+^ has been reported to be generated from the low‐temperature solution process,^32^, ^52^ and those defect sites could trap carriers, resulting in anomalous hysteresis of PSCs as well as degradation of their performances. IR spectra in **Figure** **3**a show that Ni—N vibration band at ≈526 cm^−1^ 53 is observed from the organic HTL(TPI‐6MEO)‐casted NiO*_x_* film, which is not found in pristine NiO*_x_* and organic HTL‐only film. This suggests that N atoms in organic HTLs interact with NiO*_x_*, forming Ni—N bonds that can passivate the oxygen vacancy‐induced defects in NiO*_x_*.^15^

The methoxy functional groups in those organic HTL molecules are also expected to act as Lewis bases for defect‐healing by forming Lewis adducts with undercoordinated ions. From Raman spectroscopy results of perovskite‐cast organic HTL (TPI‐6MEO) film in Figure 3b, we can clearly find peaks at 80 and 144 cm^−1^, related to Pb—O stretching,^54^ proving the formation of Lewis adducts between O atoms from electron‐donating methoxy groups of organic HTL and undercoordinated Pb ions of perovskite. To exclude the oxidation effect of perovskite that could induce Pb—O signals even without the organic HTL, the samples were characterized immediately after preparation within 1 h, and we observed that the pristine perovskite only started to generate Pb—O related signal (144 cm^−1^) after 4 h exposure to air (Figure S20, Supporting Information), which meant that Pb—O signals from the perovskite‐cast organic HTL were from the formation of Lewis adducts not from the oxidation of perovskite.

The formation of Lewis adduct was further confirmed by investigating the chemical states of Pb atoms at the interface between perovskite and organic HTL (TPI‐6MEO), obtained by XPS depth analysis of the perovskite‐cast organic HTL film. Figure 3d shows the variation of Pb 4f (from perovskite) and O 1s signal (from TPI‐6MEO) depending on the sputtering time, and the interfacial region of those two layers can be clarified by the abrupt decrease of Pb 4f signal with the increase of O 1s signal around 1.5–2.5 min of sputtering time. We especially focused on the binding energy shift of Pb 4f signals of the bulk perovskite (0.2 min sputtering time) to lower energy state at the interface (1.55 min sputtering time) (Figure 3c), and this represented the interaction of undercoordinated Pb ions in perovskite with Lewis bases in methoxy‐functionalized organic HTL at the interface,^55^ passivating possible halide vacancy defects in the perovskite.

**Figure 4:**
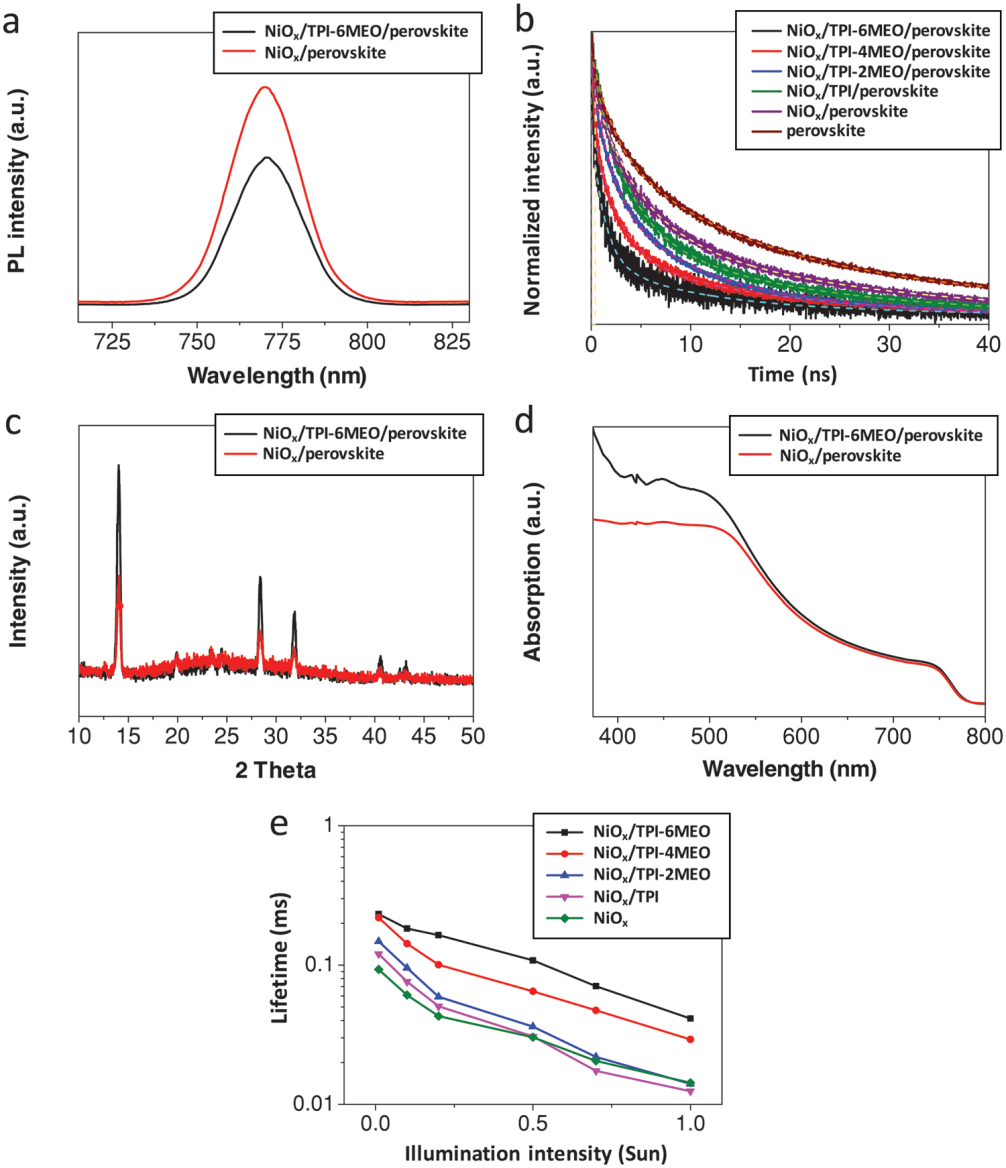
a) Steady‐state PL spectra of perovskite on NiO*_x_* layer and perovskite on TPI‐6MEO‐cast NO*_x_* layer. b) PL decay curves of perovskite on various organic HTM‐cast NiO*_x_* layer (quartz substrate, peak emission at 770 nm wavelength, and excitation at 670 nm wavelength). c) XRD patterns of perovskite on NiO*_x_* and perovskite on TPI‐6MEO‐cast NiO*_x_*. d) Absorption spectra of perovskite on NiO*_x_* and perovskite on TPI‐6MEO‐cast NiO*_x_*. e) Recombination lifetime versus light intensity plots of complete cells having various HTLs, calculated by TPV experiments.

The improved interfacial property between NiO*_x_* and perovskite with the organic HTLs is expected to provide better photocarrier extraction characteristics. **Figure** **4**a shows that the photoluminescence (PL) intensity of the perovskite on TPI‐6MEO‐cast NiO*_x_* is dramatically quenched, compared to that on bare NiO*_x_*, suggesting that interface passivation with the organic HTL is advantageous to efficiently transfer photocarriers from the perovskite to the NiO*_x_* layer. This feature was further confirmed by the decreased PL lifetimes of perovskite layers with the organic HTLs on NiO*_x_* (Figure 4b), which were calculated by convoluting decay curves using exponential functions (Table S1, Supporting Information). Figure 4b and Table S1 (Supporting Information) show that an averaged PL lifetime (τ_ave_) of perovskite on NiO*_x_* (τ_ave_ = 18.1 ns) decreases with the organic HTLs, and it is getting shorter as the number of methoxy group of organic HTL that can passivate defects increases. The gradual deepening of HOMO energy levels of organic HTLs with the addition of methoxy units (Figure 1b), explained in former section, is also expected to be beneficial to improve the hole extraction characteristics. Consequently, perovskite on TPI‐6MEO‐cast NiO*_x_*, which has the highest number of electron‐donating methoxy group, has the shortest averaged PL lifetime of 6.1 ns, showing about threefold increase in the charge extraction rate, compared to that on bare NiO*_x_*. This trend is well‐matched with *J*_sc_ variation discussed earlier.

The bifacial defect passivation of organic HTLs is also advantageous to improve the quality of perovskite layer. Figure 4c shows X‐ray diffraction (XRD) patterns of perovskite layers on pristine NiO*_x_* and organic HTL(TPI‐6MEO)‐cast NiO*_x_*, and the strong peaks at 14.5°, 28.4°, and 31.8°, which correspond to the (110), (220), and (310) planes of perovskite crystallites, are observed from both samples. Especially, the intensities of those peaks in organic HTLs‐applied perovskite are much higher than those in perovskite on bare NiO*_x_*, indicating better perovskite crystallinity with TPI‐6MEO modification. Moreover, the improved crystallinity of perovskite with organic HTL is beneficial to enhance the photon absorption as shown in Figure 4d.

The variation of charge recombination kinetics in the PSC devices with the organic HTLs was further investigated by transient photovoltage (TPV) measurement. TPV results in Figure 4e show the averaged charge recombination lifetimes of the PSCs with and without the organic HTLs, measured with an increment in *V*_oc_ (from 0 to 1.0 Sun illumination). The recombination lifetimes of all the samples decrease with the increase of light illumination intensity, because the increased carrier concentration under higher light intensity accelerates the recombination process. TPV measurement is performed at open‐circuit condition, in which charge transport effects are minimized, and therefore transient signal can be regarded as being governed by recombination only, allowing the estimation of the carrier recombination.^56^ The PSC with TPI‐6MEO has the longest recombination lifetimes in the entire range of light intensity (i.e., *V*_oc_), and the lifetimes of PSCs are prolonged as the number of methoxy unit in organic HTL increase, which indicates the retardation of charge recombination by the defect passivation and better energy level alignment with methoxy units.

### 2.4 Device Stability

**Figure 5:**
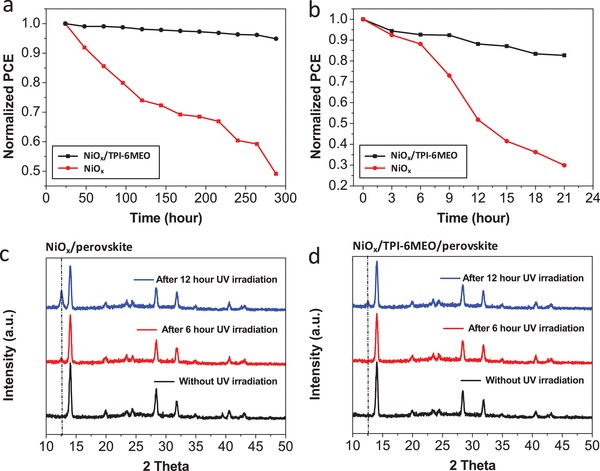
a) Stability of PSCs having NiO*_x_*‐only and TPI‐6MEO‐cast NiO*_x_* as the HTL stored in N_2_ box without encapsulation. b) Photostability test results of PSCs having NiO*_x_*‐only and TPI‐6MEO‐cast NiO*_x_* as the HTL under constant UV illumination (365 nm, 500 mW cm^−2^) without encapsulation in air. XRD pattern variation of c) perovskite on NiO*_x_* and d) perovskite on TPI‐6MEO‐cast NiO*_x_* according to continuous UV exposure time (365 nm, 500 mW cm^−2^) without encapsulation in air.

In addition to high efficiency, long‐term device stability is another crucial parameter, as it determines its suitability for commercialization. We traced the PCEs of the PSCs with and without TPI‐6MEO for 300 h of storage in N_2_ box without encapsulation. As shown in **Figure** **5**a, the PSC without organic HTL maintained about 50% of its original PCE, whereas the TPI‐6MEO‐applied PSC retained about 85% of its initial PCE within the same period. It is expected that the enhanced crystallinity of the perovskite layer with the ameliorated interfacial contact, preventing the permeation of oxygen and water into the perovskite film, improves the stability of TPI‐6MEO‐applied PSC.

Perovskite materials are known to be easily decomposed under sunlight, especially UV light. Moreover, UV irradiation accelerates the degradation of the metal oxide–perovskite interface, increasing defect sites for charge recombination. The devices with and without the TPI‐6MEO layer were exposed to continuous UV light (365 nm, 500 mW cm^−2^) in the ambient air without encapsulation, and their performances were measured every 3 h to verify the effect of the organic HTL on the UV stability of PSCs. Figure 5b shows that the PSC without organic HTL is degraded dramatically after UV light exposure for 21 h, retaining only 30% of its initial performance, but the PSC with TPI‐6MEO interlayer preserves ≈85% of its original PCE. This enhanced UV durability with TPI‐6MEO was further confirmed by XRD signal variation of perovskite films with and without TPI‐6MEO under UV irradiation. Figure 5c,d presents the XRD spectra of the perovskite films before and after UV irradiation for 6 and 12 h. Before UV light illumination, the XRD patterns of both two samples are almost identical, where the main diffraction peaks at 14.5°, 28.4°, and 31.8° are attributed to the (110), (220), and (310) planes of perovskite films. However, after 6 h UV aging, a diffraction peak centered at 12.49°, which is ascribed to the (001) diffraction peak of PbI_2_, is newly observed in the XRD patterns of perovskite film without TPI‐6MEO, and the intensity of this peak increases after 12 h UV illumination, indicating the decomposition of perovskite material into MAI and PbI_2_ under UV light. In contrast, the XRD patterns of perovskite film with TPI‐6MEO under the same UV illumination condition retain the original diffraction pattern after 6 h and show only small peak centered at 12.49° even after 12 h UV irradiation. The enhanced UV stability of PSC with TPI‐6MEO is attributed to the strong absorption of TPI‐6MEO under 400 nm wavelength light, blocking the UV light into perovskite layer, the passivated defect sites at the interface, and the improved perovskite crystallinity.

## 3 Conclusion

The defect management of perovskite and its interface with the interlayer is one of the crucial factors to achieve high‐performance and high‐stability PSCs. In this work, organic HTMs, which not only have proper optical and electrical properties for p‐i‐n PSCs without dopants but also bifacially passivate defect sites at the interfaces of perovskite and NiO*_x_* layers by forming Lewis adducts, are demonstrated. Those HTLs are also advantageous to enhance the crystallinity of following perovskite photoabsorber. Consequently, we can observe the improved charge extraction and suppressed recombination properties from the organic HTL‐applied PSCs, providing superior performances. Moreover, the UV‐blocking ability of the organic HTLs, the ameliorated interfacial property, and the improved crystallinity of perovskite significantly enhance the stability of PSCs under constant UV illumination in air without encapsulation.

## 4 Experimental Section

*Materials and Characterization: N*,*N*,*N*′,*N*′‐tetraphenylbenzidine, phosphorus oxychloride (POCl_3_), *N*,*N*′‐dimethylformamide (DMF) anhydrous, aniline, 4‐methoxyaniline, benzil, 4,4′‐dimethoxybenzil, ammonium acetate, and glacial acetic acid were purchased from Sigma Aldrich and used without further purification. All glasswares, syringes, magnetic stirrer bars, and needles were thoroughly dried before use. Reactions were monitored using thin layer chromatography (TLC) with TLC plates (silica gel 60 F254, Merck Co.). Silica gel column chromatography was performed with silica gel 60 (particle size 0.0063–0.200 mm, Merck). ^1^H NMR and ^13^C NMR spectra were obtained from Bruker (Fourier 300 MHz) and Oxford (Fourier 400 MHz) NMR spectrometers, and the chemical shifts (δ) and coupling constant (*J*) were expressed in ppm and Hertz, respectively. ESI‐MS spectra were obtained on a UHPLC/tandem mass spectrometer (1290 infinity II/Qtrap 6500). Absorption spectra were monitored by UV‐2600 UV–vis spectrophotometer (SHIMADZU) in the wavelength range 200–900 nm. Cyclic voltammetry analysis was performed on a potentiostat/galvanostat model 273A (Princeton Applied Research) for obtaining the potential. A supporting electrolyte was made to 0.1 m tetra‐*n*‐butylammonium tetrafluoroborate solution in dichloromethane. Parameters were set to scan rate of 100 mV s^−1^ and vertex potential of 2.5 V.

*Device Fabrication*: PSC devices were fabricated on patterned indium‐doped tin oxide (ITO) coated glass. ITO was etched with 35% HCl and zinc powder. These substrates were cleaned using ultrasonication in acetone, isopropyl alcohol (IPA), and deionized water for about 3 min, respectively. Substrates were then treated by oxygen plasma for the enhanced wettability of the following HTM solution. NiO*_x_* nanoparticle solution (20 mg mL^−1^) was spun on the ITO layer (2000 rpm for 20 s) and annealed at 100 °C for 10 min. NiO*_x_* nanoparticle synthesis procedures are described elsewhere.^57^ Triphenylamine‐imidazole‐based HTMs were dissolved in chloroform at a concentration of 20 mg mL^−1^, and then spin‐cast on the NiO*_x_* layer at 2000 rpm for 20 s. CH_3_NH_3_PbI_3_ perovskite solution was prepared by dissolving PbI_2_ (Sigma Aldrich) and CH_3_NH_3_I (1:1 molar ratio) in the solvent mixture of DMF and dimethylsulfoxide (DMSO) (9:1 v/v) for a total concentration of 1.6 m in a N_2_ atmosphere. The solution was stirred at room temperature for at least 2 h before being used, then filtered via polytetrafluoroethlyene (PTFE) filter (0.45 µm). The perovskite layer was formed onto the HTM by a one‐step spin‐casting process at 4000 rpm for 25 s. After 9 s, the substrate was treated with ether (0.5 mL) by drop‐casting. The substrate was annealed on a hot plate at 100 °C for 10 min. PCBM (20 mg mL^−1^ in chlorobenzene) was spin‐cast on the perovskite layer. BCP (0.5 mg mL^−1^ in ethanol) solution was prepared and spun at 5000 rpm for 60 s, and then dried 10 min. On the last stage, samples were transferred into a thermal evaporator and Ag (100 nm) was deposited at a pressure of 5 × 10^−6^ torr giving a following device configuration: ITO/NiO*_x_*/organic HTL/CH_3_NH_3_PbI_3_/PCBM/BCP/Ag. The hole‐only devices to estimate the hole mobility values of organic HTLs were fabricated by utilizing MoO_3_ having high work function to block the injection of electrons from the Ag electrode (ITO/NiO*_x_*/organic HTL/MoO_3_/Ag).

*Device Characterization*: A solar simulator (PEC‐L01, Peccell Technologies, Inc.) with AM 1.5 G illumination provides 100 mW cm^−2^ of illumination on the PV cells. The intensity was calibrated using a NREL‐certified Si photodiode, equipped with an infrared cutoff filter (KG5) to reduce spectral mismatch. *J*–*V* characteristics were obtained using an Ivium Technology Ivium CompactStat by scanning at a 0.05 V s^−1^ scan rate. The EQE was measured at short‐circuit condition using an ABET Technology 10500 solar simulator as the light source and a SPECTRO Mmac‐200 as the light solution.

*FTIR, Raman, XRD, and XPS Characterization*: FTIR spectra were recorded by a Nicolet 5700 instrument (Thermofisher Scientific, USA) from 4000 to 400 cm^−1^ with a resolution of 4 cm^−1^. Samples have the following configurations: Si wafer/NiO*_x_*, Si wafer/TPI‐6MEO, and Si wafer/NiO*_x_*/TPI‐6MEO. Raman spectra were obtained by a high‐resolution Raman spectrometer (LabRam HR Evolution, HORIBA, Japan) with a pumped laser of 532 nm and the resolution of 600 gr mm^−1^. Raman spectra were measured for 20 s by a 100× objective, resulting in a laser spot diameter less than 1 µm on the sample. Raman samples have the following configurations: glass/TPI‐6MEO/perovskite and glass/perovskite. XRD patterns were collected via an Ultima III (Rigaku) diffractometer using copper Kα radiation. X‐ray pattern was measured by step scanning at angular intervals of 0.08° from 10° to 90°. 2θ scans were obtained from samples having the following configurations: glass/NiO*_x_*/perovskite and glass/NiO*_x_*/TPI‐6MEO/perovskite). XPS measurements were carried out using a PHI 5000 VersaProbe spectrometer equipped with an Al Kα X‐ray source (1486.6 eV). An Ar^+^ ion gun with a 2 kV beam voltage in a 2 × 2 mm raster area (yielding an equivalent sputtering rate of 8 nm min^−1^ of SiO_2_) was used to XPS depth profiling. All binding energies in the XPS data were calibrated with reference to the C−C bond in the C 1s. The sample for XPS measurement has a glass/TPI‐6MEO/perovskite configuration.

*Time‐Resolved Photoluminescence (TRPL) and TPV*: TRPL curves were recorded using a commercial TCSPC system (FluoTime 200, PicoQuant). Samples were excited by using a picosecond diode laser of 670 nm (LDH‐P‐C‐670, PicoQuant) with a repetition rate of 4 MHz. The emitted PL signal was accumulated via a fast photomultiplier tube (PMT) detector (PMA 182, PicoQuant) with a magic angle (54.7°) arrangement. The incident angle of excitation pulse was set to be about 30° with respect to the sample. The resulting instrumental response function was 160 ps in full width at half maximum. The PL decays were measured at the emission peak (770 nm) for perovskite. In addition, a cutoff filter (FF01‐692 nm, Semrock) was applied to block the scattering. The transient photovoltage measurement was conducted by a nanosecond OPO laser system (INDI‐40‐10, Spectra‐Physics) with Nd:YAG laser and a background illumination from Xe lamp (LS‐150‐XE ABET). An attenuated laser pulse at 550 nm laser pulse with a pulse width of 120 fs was used as a small perturbation to the background illumination for generating an additional amount of charge on the devices. The laser‐pulse‐induced photovoltage variation was smaller than 3% of the *V*_oc_ not exceeding 20 mV produced by the background illumination. The device was connected to a digital oscilloscope (DSO‐X 3054A, Agilent) with BNC cables, and the input impedance of the oscilloscope was set to 1 MΩ to form the open‐circuit conditions. The bias light intensity was adjusted by neutral density filters (NDC‐100C‐4M) for various *V*_oc_ values. The initial light intensity from the Xe lamp was modified using power meter to be equivalent to 1 Sun (100 mA cm^−2^).

## Conflict of Interest

The authors declare no conflict of interest.

## Supporting information

Supplementary
