# Supplementary material for: Bifacial Passivation of Organic Hole Transport Interlayer for NiOx‐Based p‐i‐n Perovskite Solar Cells
Source: Adv Sci (Weinh). 2019 Jan 29;6(6):1802163. doi: 10.1002/advs.201802163 (PMC6425451; doi:10.1002/advs.201802163)
Supplement: Supplementary file 1 — Supplementary [file ADVS-6-1802163-s001.pdf]

Copyright WILEY-VCH Verlag GmbH & Co. KGaA, 69469 Weinheim, Germany, 2019.

ADVANCED SCIENCE Open Access

Supporting Information

for *Adv. Sci.,* DOI: 10.1002/advs.201802163

Bifacial Passivation of Organic Hole Transport Interlayer for NiO_x-Based p-i-n Perovskite Solar Cells

Zijia Li, Bong Hyun Jo, Su Jin Hwang, Tae Hak Kim, Sivaraman Somasundaram, Eswaran Kamaraj, Jiwon Bang, Tae Kyu Ahn, Sanghyuk Park, and Hui Joon Park**

Copyright WILEY-VCH Verlag GmbH & Co. KGaA, 69469 Weinheim, Germany, 2019.

Supporting Information

Bifacial Passivation of Organic Hole Transport Interlayer for NiO_x-Based p-i-n Perovskite Solar Cells

Zijia Li, Bong Hyun Jo, Su Jin Hwang, Tae Hak Kim, Sivaraman Somasundaram, Eswaran Kamaraj, Jiwon Bang, Tae Kyu Ahn, Sanghyuk Park and Hui Joon Park**

Z. Li, B. H. Jo, Prof. T. K. Ahn
Department of Energy Science
Sungkyunkwan University
Suwon 16419, Republic of Korea

S. J. Hwang, S. Somasundaram, E. Kamaraj, S. Park
Department of Chemistry,
Kongju National University
Kongju 32588, Republic of Korea
E-mail: spark0920@kongju.ac.kr

J. Bang
Nano Convergence Materials Center
Korea Institute of Ceramic Engineering & Technology
Jinju 52851, Republic of Korea

T. H. Kim, Prof. H. J. Park
Department of Energy Systems Research
Ajou University
Suwon 16499, Republic of Korea
E-mail: huijoon@ajou.ac.kr

Prof. H. J. Park
Department of Electrical and Computer Engineering
Ajou University
Suwon 16499, Republic of Korea

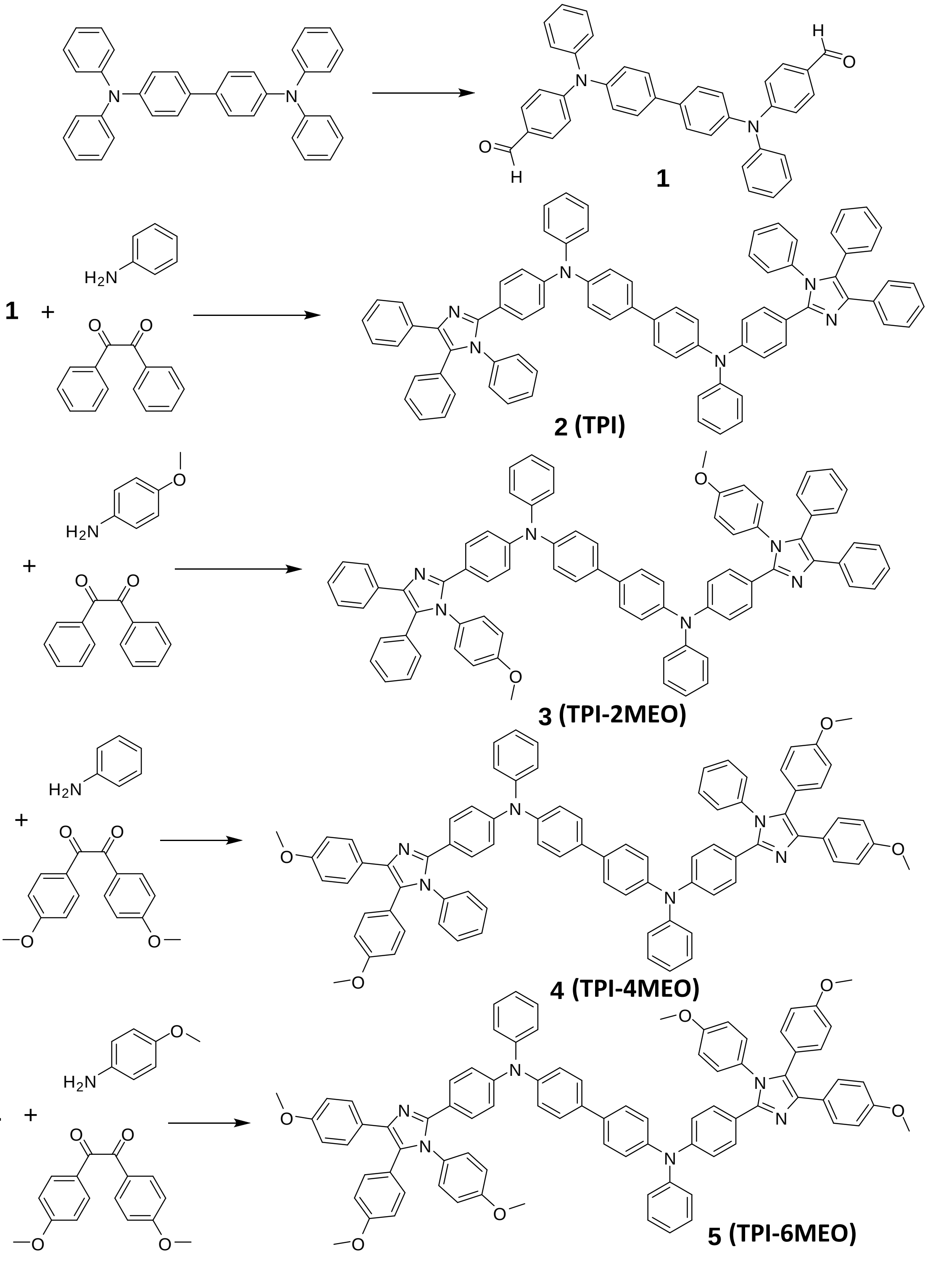

Figure S1. Synthetic routes of the triphenylamine-imidazole-based hole transporting molecules.

1. Materials synthesis

4,4'-([1,1'-biphenyl]-4,4'-diylbis(phenylazanediyl))dibenzaldehyde (1):

Under nitrogen atmosphere, $POCl_3$ (40.9 mmol) in anhydrous DMF (20.0 mL) was stirred at 0℃ for 1h. *N,N,N',N'*-tetraphenylbenzidine (2.00 g, 4.09 mmol) was slowly added into the reaction flask. The reaction mixture was stirred at 95℃ for 12h. After the completion of reaction, the mixture was cooled to room temperature and poured into copious amount of ice water, then neutralized with sodium bicarbonate. The product mixture was extracted with CH_2Cl_2 together with brine, and dried over anhydrous $MgSO_4$. The crude product was purified by silica gel column chromatography (ethyl acetate: hexane = 1: 7 v/v) to afford compound **1** (1.2g, 54%) ^1H NMR (400 MHz, $CDCl_3$) δ 9.83 (s, 2H), 7.71 (d, J = 8.7 Hz, 4H), 7.55 (d, J = 8.5 Hz, 4H), 7.39 – 7.34 (t, 4H), 7.21 (dt, J = 15.2, 7.7 Hz, 10H), 7.08 (d, J = 8.7 Hz, 4H). ^{13}C NMR ($CDCl_3$) δ 190.40 (s), 153.13 (s), 146.05 (s), 145.42 (s), 136.65 (s), 131.30 (s), 129.79 (s), 129.37 (s), 127.95 (s), 126.37 (s), 126.18 (s), 125.25 (s), 119.78 (s). Elemental anal. Calcd. for $C_{38}H_{28}N_2O_2$: C, 83.80; H, 5.18; N, 5.14; O, 5.88. Found: C, 83.56; H, 5.18; N, 5.08; O, 5.18.

General Procedure for the synthesis of Imidazole derivatives:

1.00 g of dibenzaldehyde compound 1 (1.84 mmol, 1 eq.) and aniline derivative (3.86 mmol, 2 eq.) in glacial acetic acid (60 mL) were stirred at room temperature for 30 minutes. After the mixture changed to dark red, benzil derivative (3.86 mmol, 2 eq.) and ammonium acetate (9.18 mmol, 6 eq.) were added and stirred at 120℃ for 12h. After the completion of reaction, the mixture was cooled down to room temperature. Cooled reaction mixture was poured into 600 ml of water and neutralized with sodium bicarbonate. The crude product was filtered and precipitated successively in ethyl acetate/hexane and dichloromethane/methanol. The crude

product was purified by silica gel column chromatography (ethyl acetate : hexane = 1: 10 v/v) to afford corresponding imidazole compounds.

N^4,$N^{4'}$-diphenyl-N^4,$N^{4'}$-bis(4-(1,4,5-triphenyl-1H-imidazol-2-yl)phenyl)-[1,1'-biphenyl]-4,4'-diamine (2, TPI):

Following the general procedure for the synthesis of imidazole derivatives, compound 1, aniline, and benzil were used for the preparation of compound 2. Yield : 1.0 g, 52%. ^{1}H NMR (300 MHz, $CDCl_3$): δ (ppm) 7.63-7.61 (d, 4H), 7.46-7.43 (d, 4H), 7.33-7.20 (m, 26H), 7.15-7.03 (m, 18H), 6.97-6.95 (d, 4H). ^{13}C NMR ($CDCl_3$) δ 131.11 (s), 129.66 (s), 129.31 (s), 129.04 (d, J = 2.2 Hz), 128.44 (d, J = 6.3 Hz), 128.26 (d, J = 5.9 Hz), 128.12 (s), 127.37 (s), 124.94 (s), 124.70 (s), 124.11 (s), 123.42 (s), 122.32 (s). ESI-MS (m/z): 1077.5(M^+). Elemental anal. Calcd for $C_{78}H_{56}N_6$: C, 86.96; H, 5.24; N, 7.80. Found: C, 87.11; H, 5.36; N, 7.53.

N^4,$N^{4'}$-bis(4-(1-(4-methoxyphenyl)-4,5-diphenyl-1H-imidazol-2-yl)phenyl)-N^4,$N^{4'}$-diphenyl-[1,1'-biphenyl]-4,4'-diamine (3, TPI-2MEO):

Following the general procedure for the synthesis of imidazole derivatives, compound **1,** *p*-anisidine(4-methoxyaniline), and benzil were used for the preparation of compound **3**. Yield: 1.1 g, 53%. ^{1}H NMR (300 MHz, $CDCl_3$): δ (ppm) 7.59 (dd, J = 8.2, 1.4 Hz, 4H), 7.44 (d, J = 8.6 Hz, 4H), 7.31 (t, J = 7.6 Hz, 6H), 7.24 – 7.17 (m, 12H), 7.15 – 6.93 (m, 24H), 6.77 (d, J = 8.9 Hz, 4H), 3.77 (s, 6H). ^{13}C NMR ($CDCl_3$): δ (ppm) 159.139, 147.591, 147.212, 146.909, 146.294, 135.205, 131.169, 130.877, 130.823, 130.009, 129.601, 129.514, 129.340, 128.321, 128.125, 127.840, 127.373, 126.515, 124.993, 124.763, 123.425, 122.384, 114.211, 55.359. ESI-MS (m/z): 1137.6(M^+). Elemental anal. Calcd for $C_{80}H_{60}N_6O_2$: C, 84.48; H, 5.32; N, 7.39; O, 2.81. Found: C, 84.40; H, 5.44; N, 7.52; O, 2.64.

N^4,$N^{4'}$-bis(4-(4,5-bis(4-methoxyphenyl)-1-phenyl-1H-imidazol-2-yl)phenyl)-N^4,$N^{4'}$-diphenyl-[1,1'-biphenyl]-4,4'-diamine (4, TPI-4MEO):

Following the general procedure for the synthesis of imidazole derivatives, compound **1,** aniline, and anisil (4,4'-dimethoxybenzil) were used for the preparation of compound **4**. Yield: 1.0 g, 46%. ^{1}H NMR (300 MHz, $CDCl_3$): δ (ppm) 7.56-7.53 (d, 4H), 7.43-7.40 (d, 4H), 7.30-7.22 (m, 14H), 7.10-7.01 (m, 18H), 6.95-6.92 (d, 4H), 6.81-6.78 (d, 4H), 6.75-6.72 (d, 4H), 3.77 (s, 6H), 3.75 (s, 12H). ^{13}C NMR ($CDCl_3$): δ (ppm) 159.120, 158.376, 147.560, 147.206, 146.367, 146.293, 137.621, 137.422, 135.179, 132.400, 129.654, 129.586, 129.337, 129.063, 128.561, 128.498, 128.200, 127.395, 127.261, 124.963, 124.731, 123.407, 122.992, 122.412, 113.827, 113.613, 55.197, 55.123. Elemental anal. Calcd for $C_{82}H_{64}N_6O_4$: C, 82.25; H, 5.39; N, 7.02; O, 5.34. Found: C, 82.36; H, 5.39; N, 6.97; O, 5.28.

N^4,$N^{4'}$-diphenyl-N^4,$N^{4'}$-bis(4-(1,4,5-tris(4-methoxyphenyl)-1H-imidazol-2-yl)phenyl)-[1,1'-biphenyl]-4,4'-diamine (5, TPI-6MEO):

Following the general procedure for the synthesis of imidazole derivatives, compound **1,** p-anisidine(4-methoxyaniline), and anisil (4,4'-dimethoxybenzil) were used for the preparation of compound **5**. Yield: 1.3g, 57%. ^{1}H NMR (300 MHz, $CDCl_3$): δ (ppm) 7.57-7.54 (d, 4H), 7.46-7.35 (d, 4H), 7.32-7.25 (m, 8H), 7.13-6.95 (m, 22H), 6.83-6.76 (m, 12H), 3.79 (s, 18H). ^{13}C NMR ($CDCl_3$): δ (ppm) 159.100, 146.263, 132.406, 129.801, 129.616, 129.523, 129.334, 128.525, 127.408, 125.005, 124.775, 123.432, 122.311, 114.220, 113.829, 113.600, 55.362, 55.187, 55.124. ESI-MS (m/z): 1257.5(M^+). Elemental anal. Calcd for $C_{84}H_{68}N_6O_6$: C, 80.23; H, 5.45; N, 6.68; O, 7.63. Found: C, 80.37; H, 5.42, N, 6.61; O, 7.60.

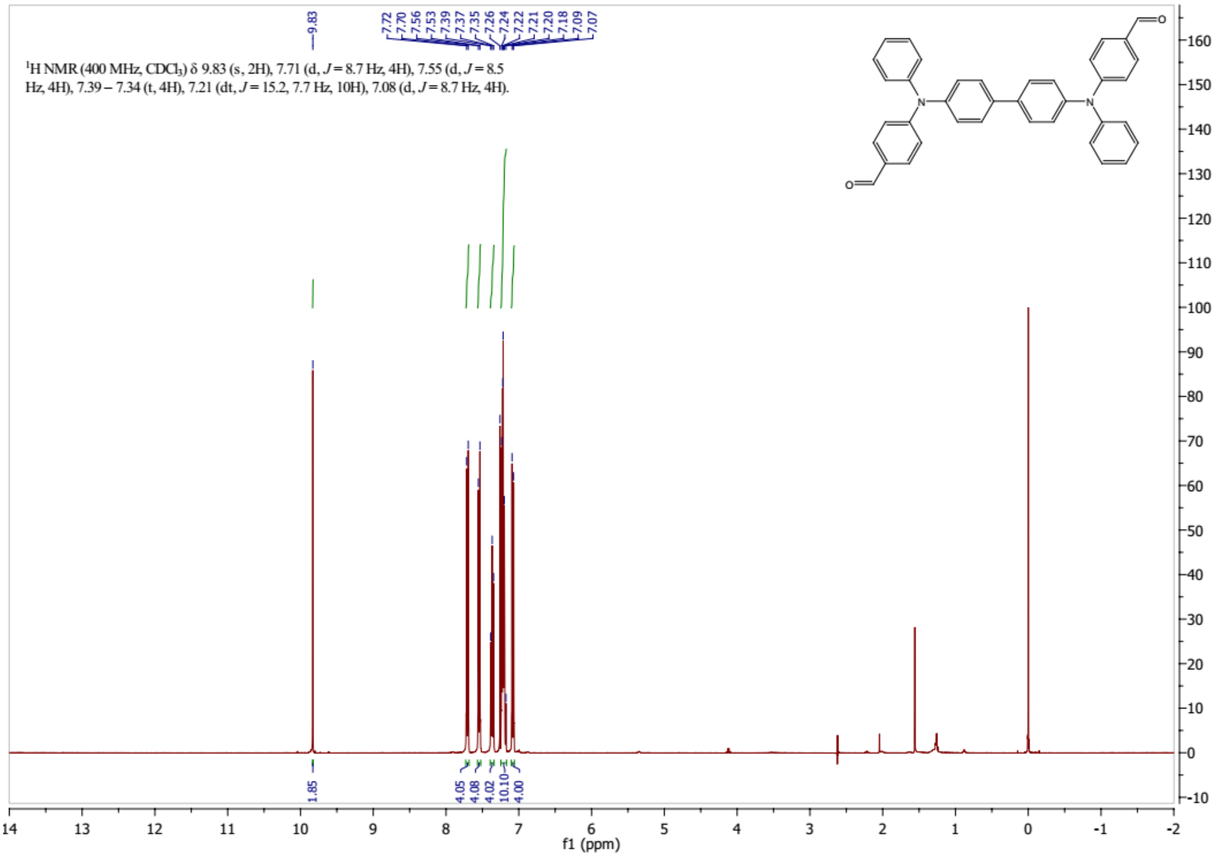

Figure S2. ^{1}H NMR (400 MHz, $CDCl_3$) of 4,4'-([1,1'-biphenyl]-4,4'-diylbis(phenylazanediyl))dibenzaldehyde (1).

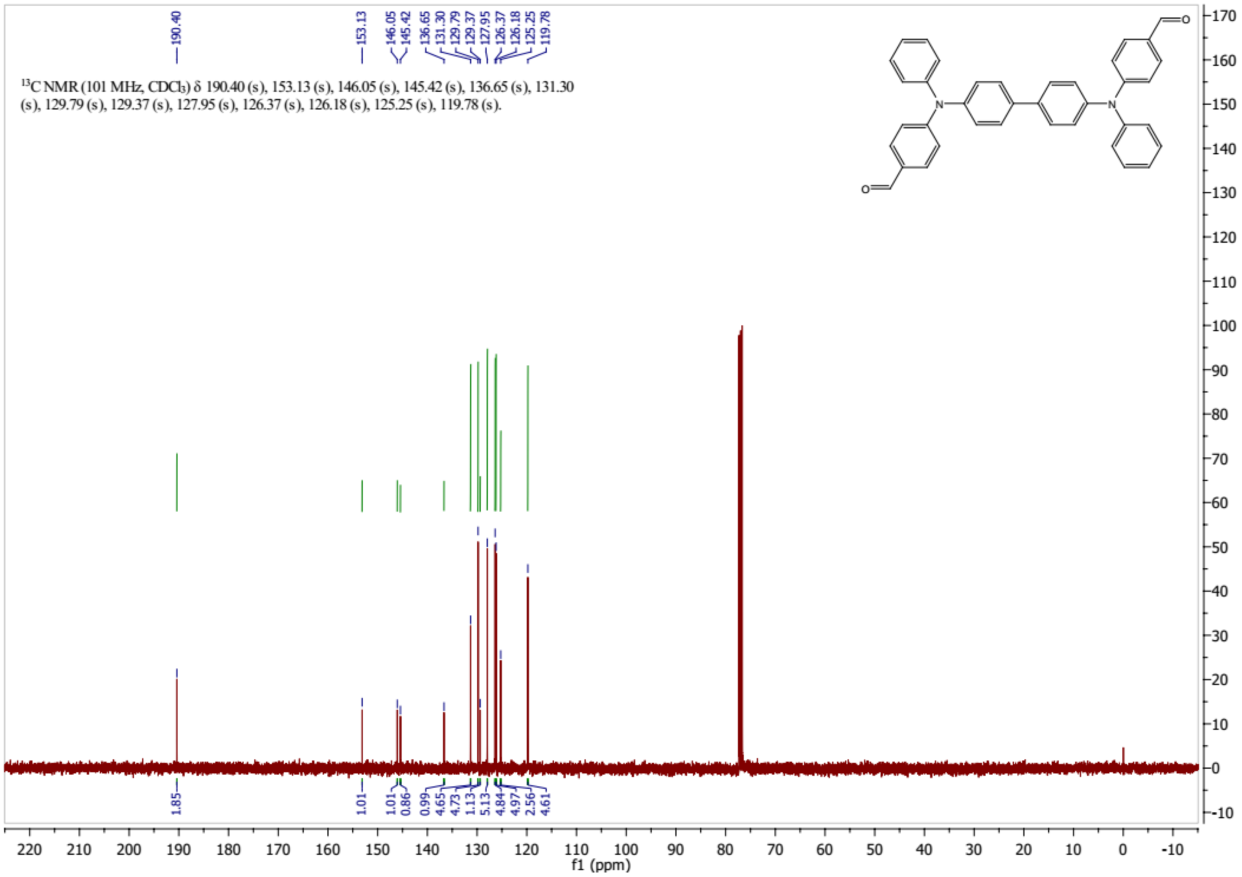

Figure S3. ^{13}C NMR ($CDCl_3$) of 4,4'-([1,1'-biphenyl]-4,4'-diylbis(phenylazanediyl))dibenzaldehyde (1).

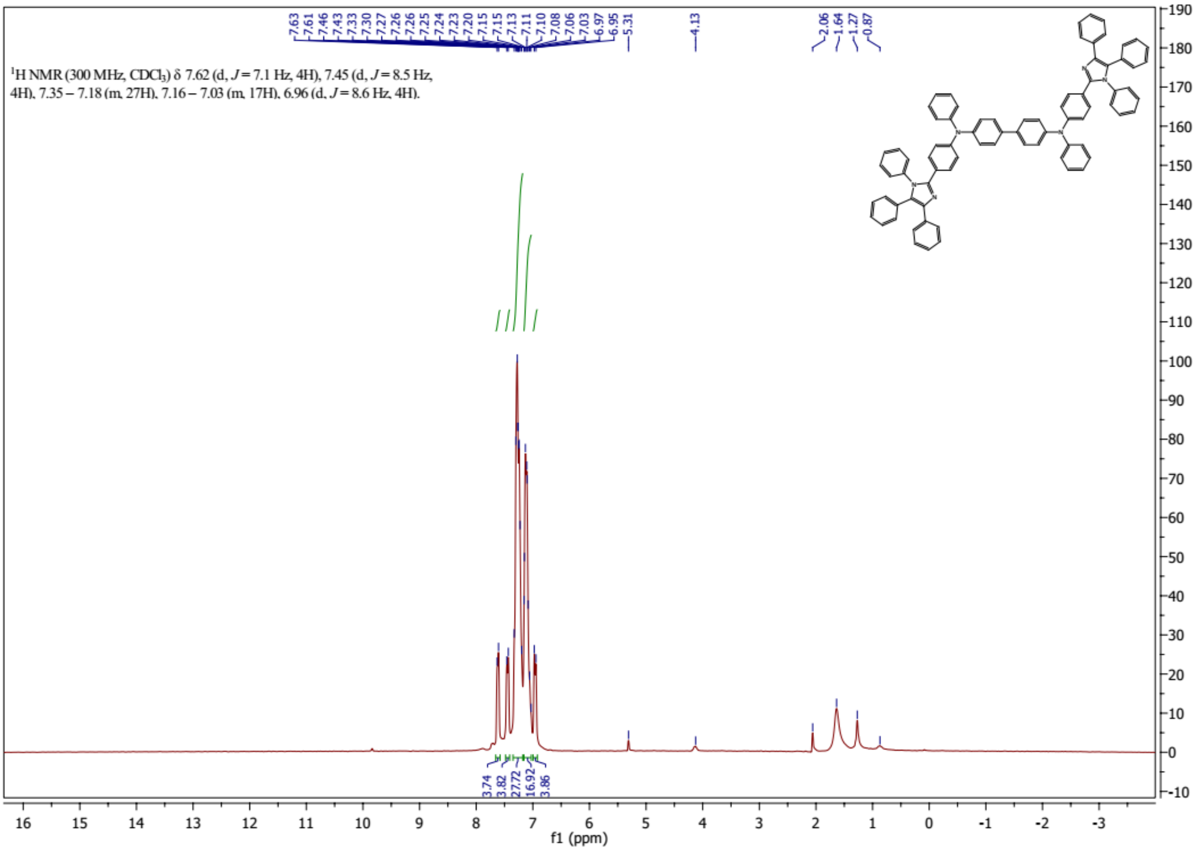

Figure S4. ^{1}H NMR (400 MHz, $CDCl_3$) of N^{4},N$^{4'}$-diphenyl-N^{4},N$^{4'}$-bis(4-(1,4,5-triphenyl-1H-imidazol-2-yl)phenyl)-[1,1’-biphenyl]-4,4’-diamine (2, TPI).

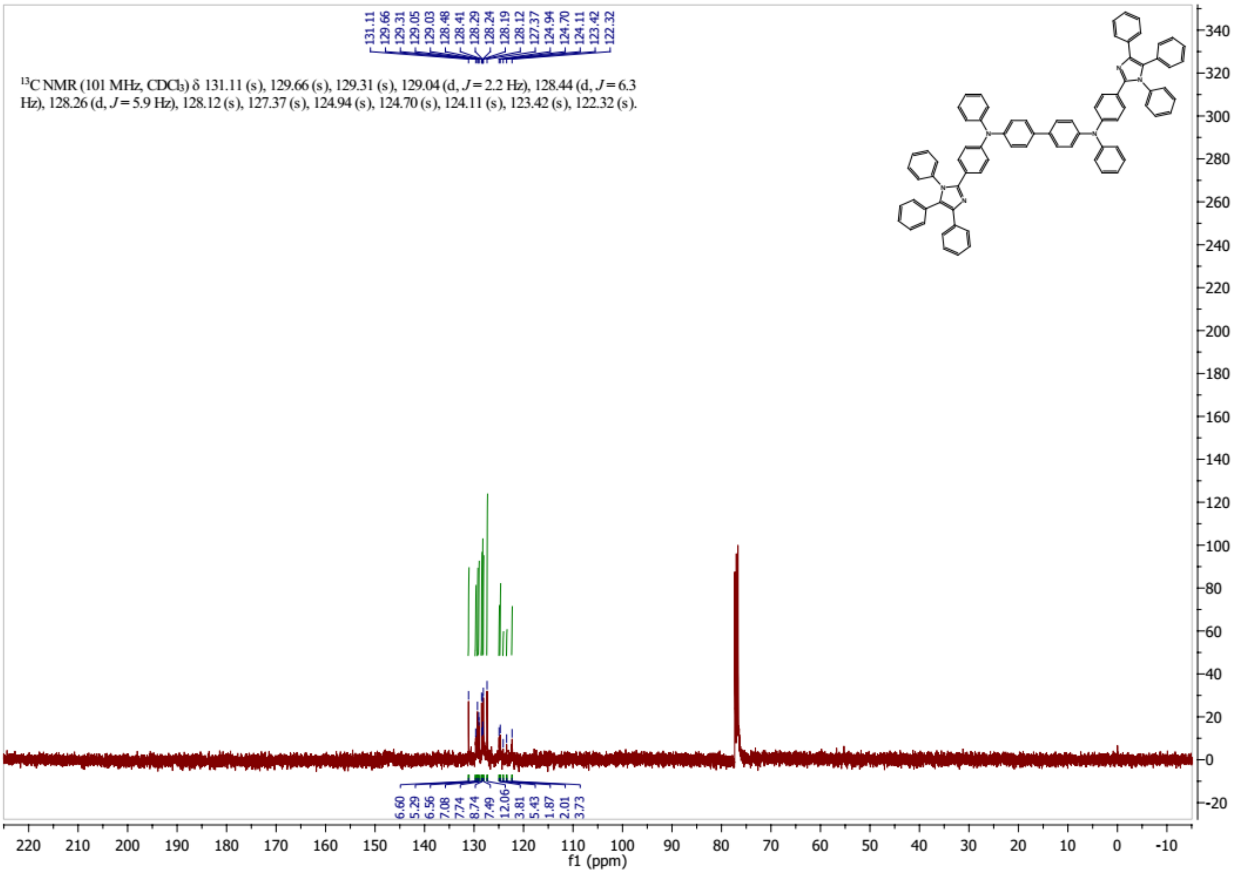

Figure S5. ^{13}C NMR ($CDCl_3$) of N^{4},N$^{4'}$-diphenyl-N^{4},N$^{4'}$-bis(4-(1,4,5-triphenyl-1H-imidazol-2-yl)phenyl)-[1,1’-biphenyl]-4,4’-diamine (2, TPI).

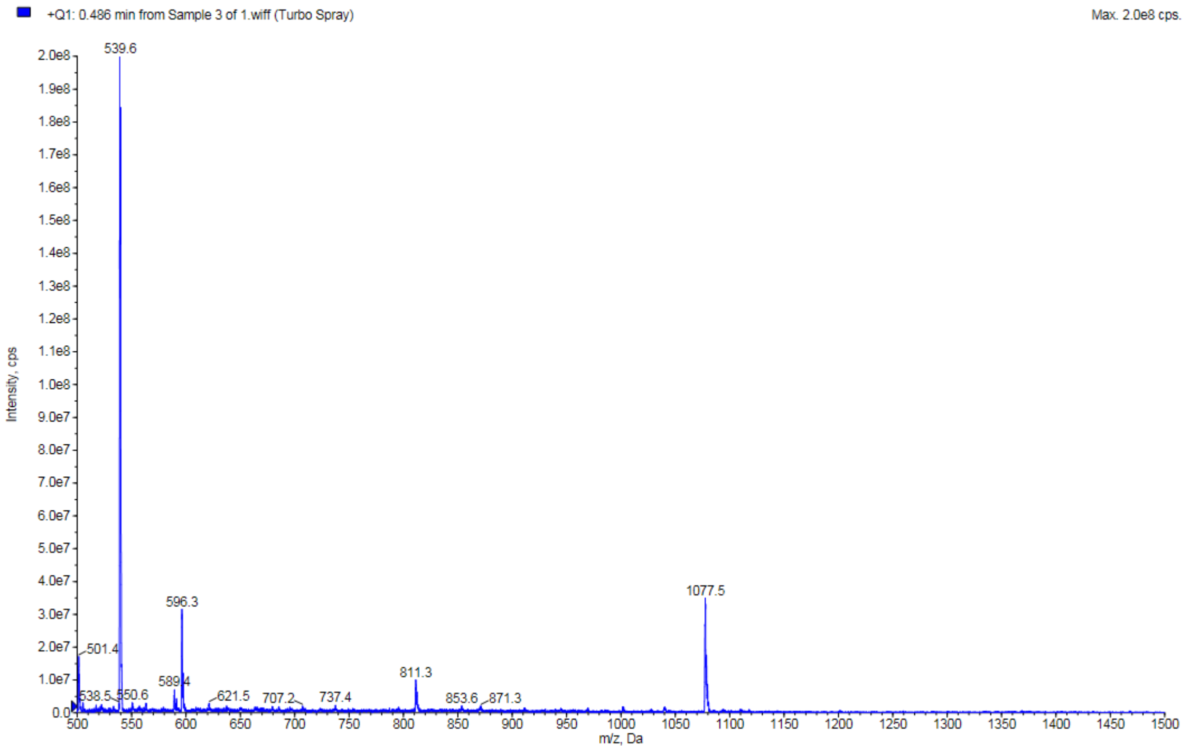

Figure S6. ESI-MS results of N[4],N[4']-diphenyl-N[4],N[4']-bis(4-(1,4,5-triphenyl-1H-imidazol-2-yl)phenyl)-[1,1'-biphenyl]-4,4'-diamine (2, TPI, M_W=1077.32).

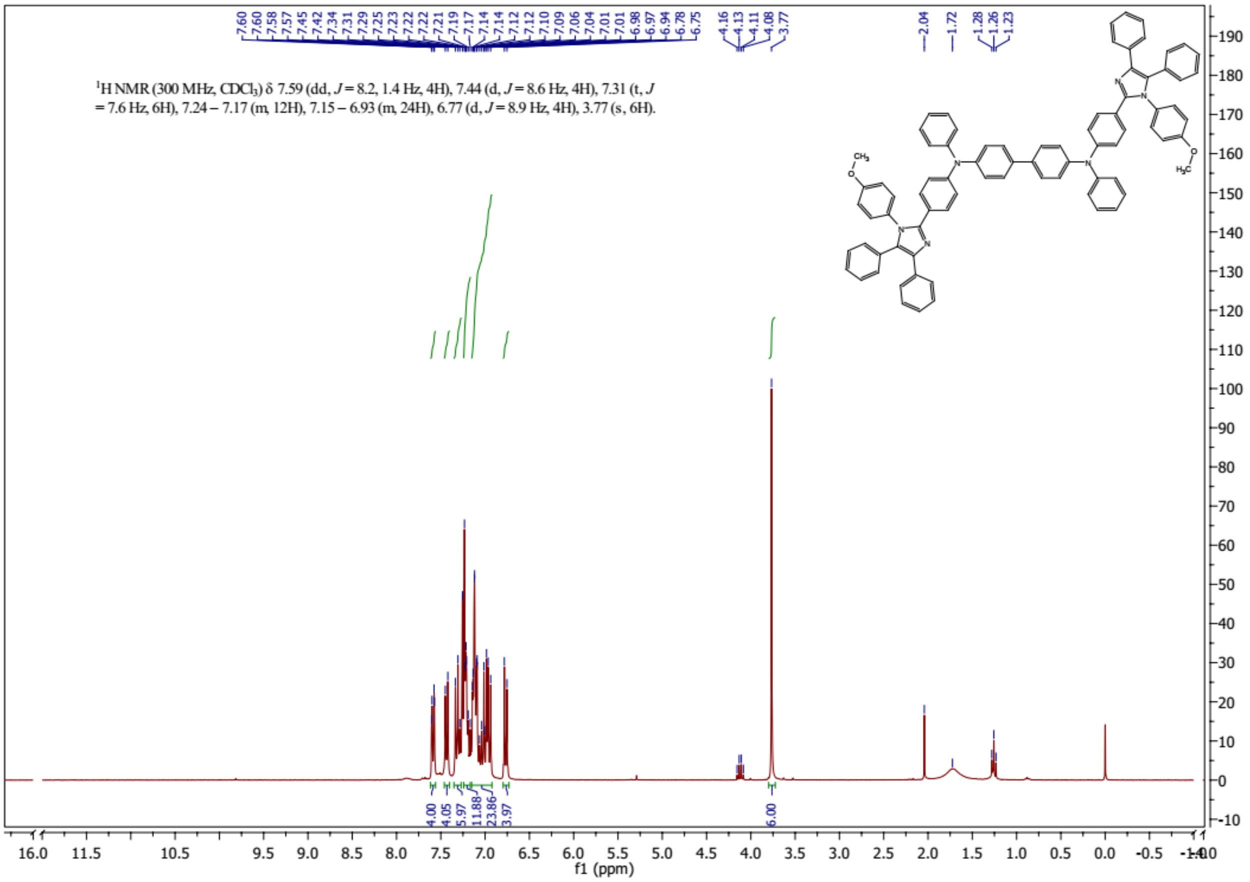

Figure S7. ^{1}H NMR (300 MHz, $CDCl_3$) of N4,N4’-bis(4-(1-(4-methoxyphenyl)-4,5-diphenyl-1H-imidazol-2-yl)phenyl)-N4,N4’-diphenyl-[1,1’-biphenyl]-4,4’-diamine (3, TPI-2MEO).

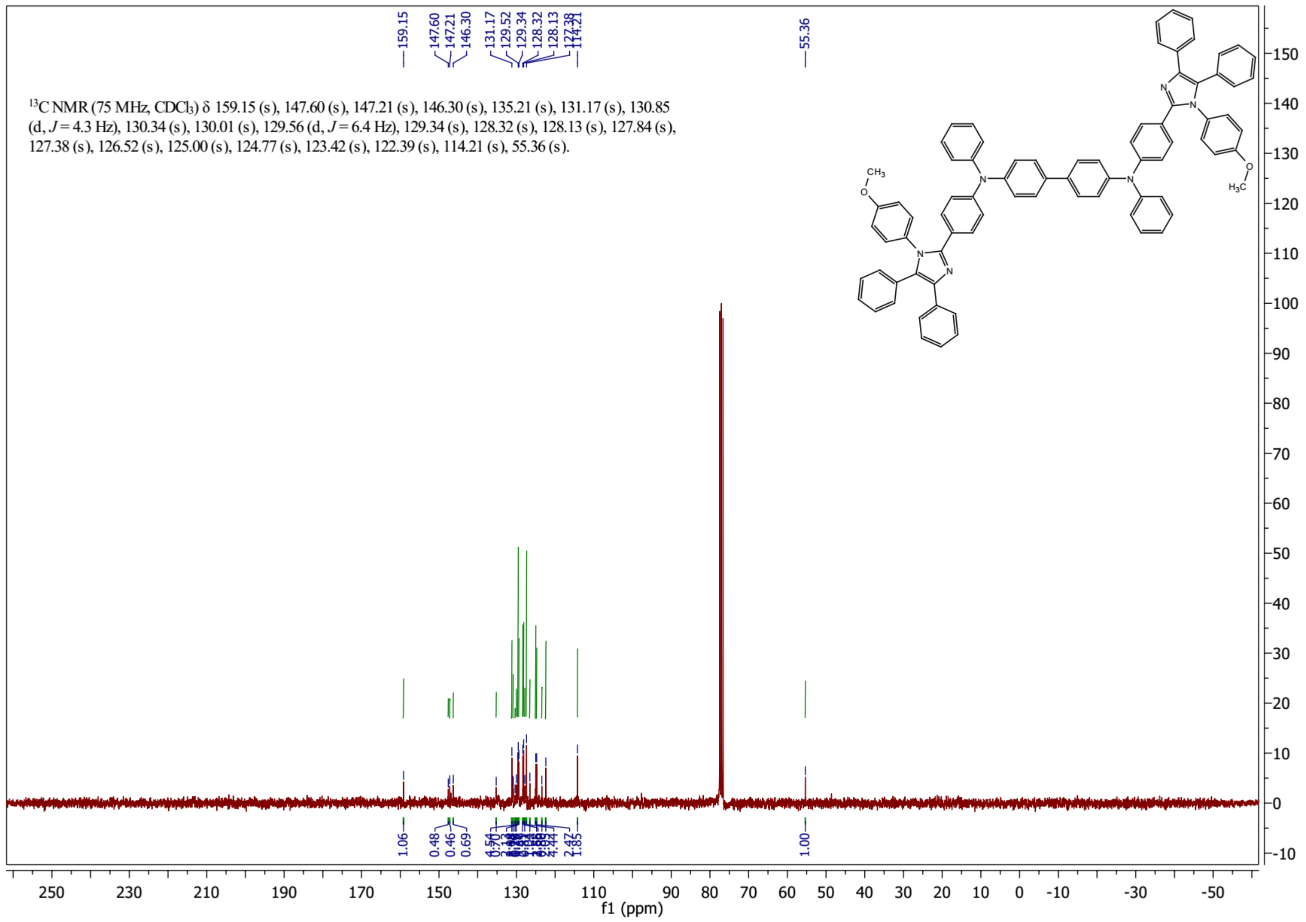

Figure S8. ^{13}C NMR ($CDCl_3$) of N4,N4'-bis(4-(1-(4-methoxyphenyl)-4,5-diphenyl-1H-imidazol-2-yl)phenyl)-N4,N4'-diphenyl-[1,1'-biphenyl]-4,4'-diamine (3, TPI-2MEO).

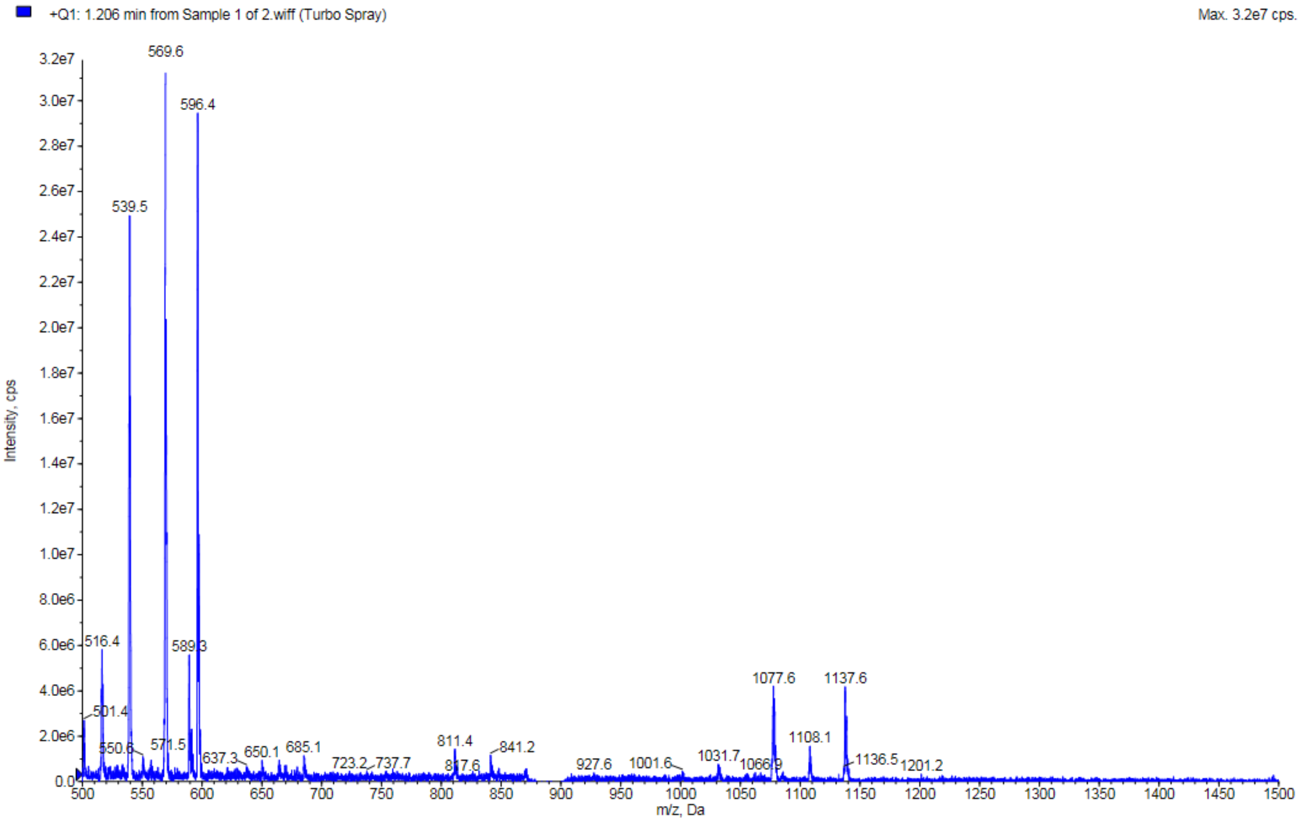

Figure S9. ESI-MS results of N4,N4'-bis(4-(1-(4-methoxyphenyl)-4,5-diphenyl-1H-imidazol-2-yl)phenyl)-N4,N4'-diphenyl-[1,1'-biphenyl]-4,4'-diamine (3, TPI-2MEO, M_W=1137.37).

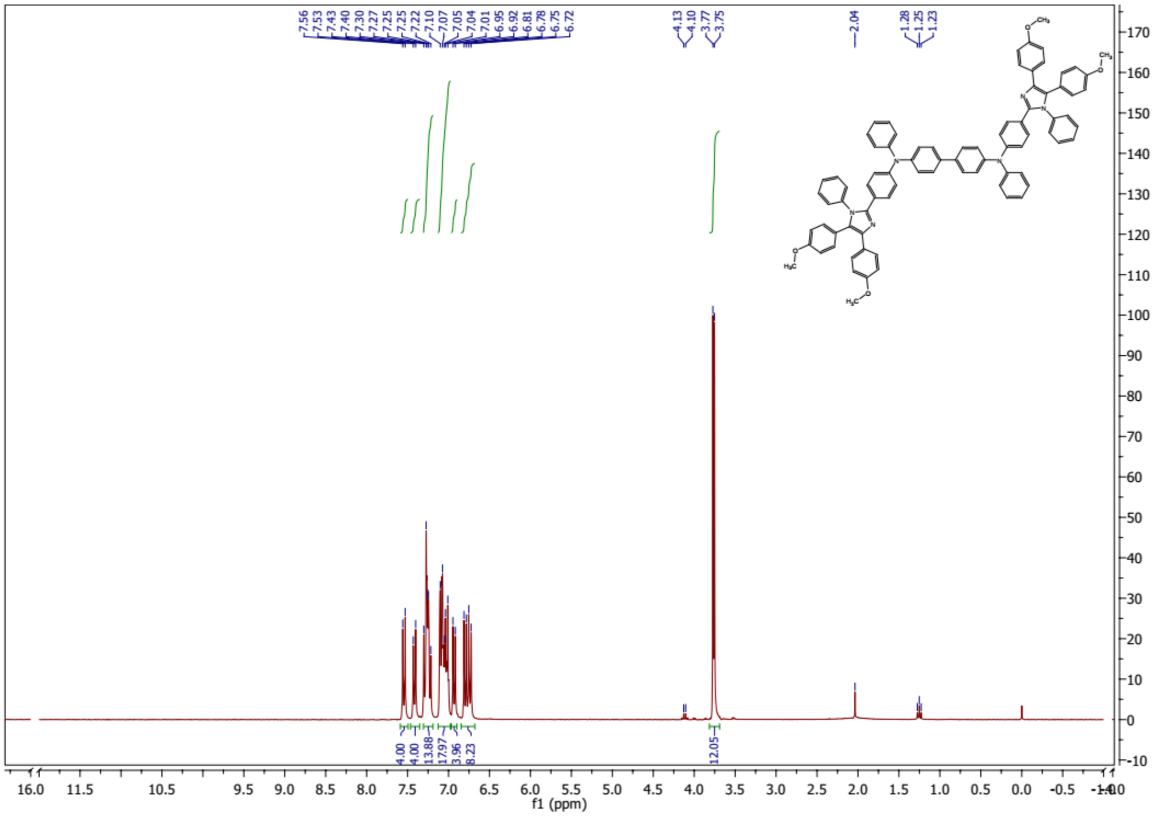

Figure S10. ^{1}H NMR (300 MHz, $CDCl_3$) of N4,N4'-bis(4-(4,5-bis(4-methoxyphenyl)-1-phenyl-1H-imidazol-2-yl)phenyl)-N4,N4'-diphenylbiphenyl-4,4'-diamine (4, TPI-4MEO).

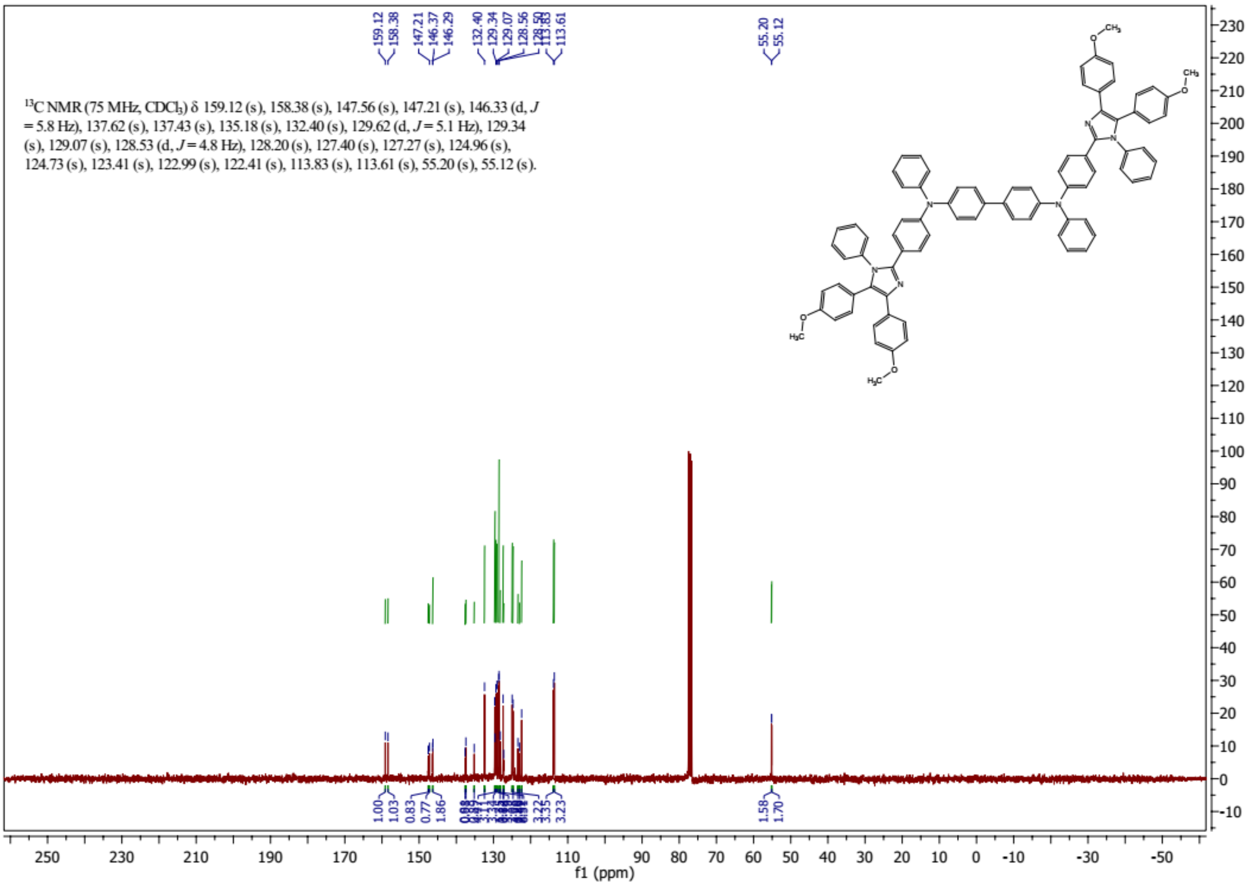

Figure S11. ^{13}C NMR ($CDCl_3$) of N4,N4'-bis(4-(4,5-bis(4-methoxyphenyl)-1-phenyl-1H-imidazol-2-yl)phenyl)-N4,N4'-diphenylbiphenyl-4,4'-diamine (4, TPI-4MEO).

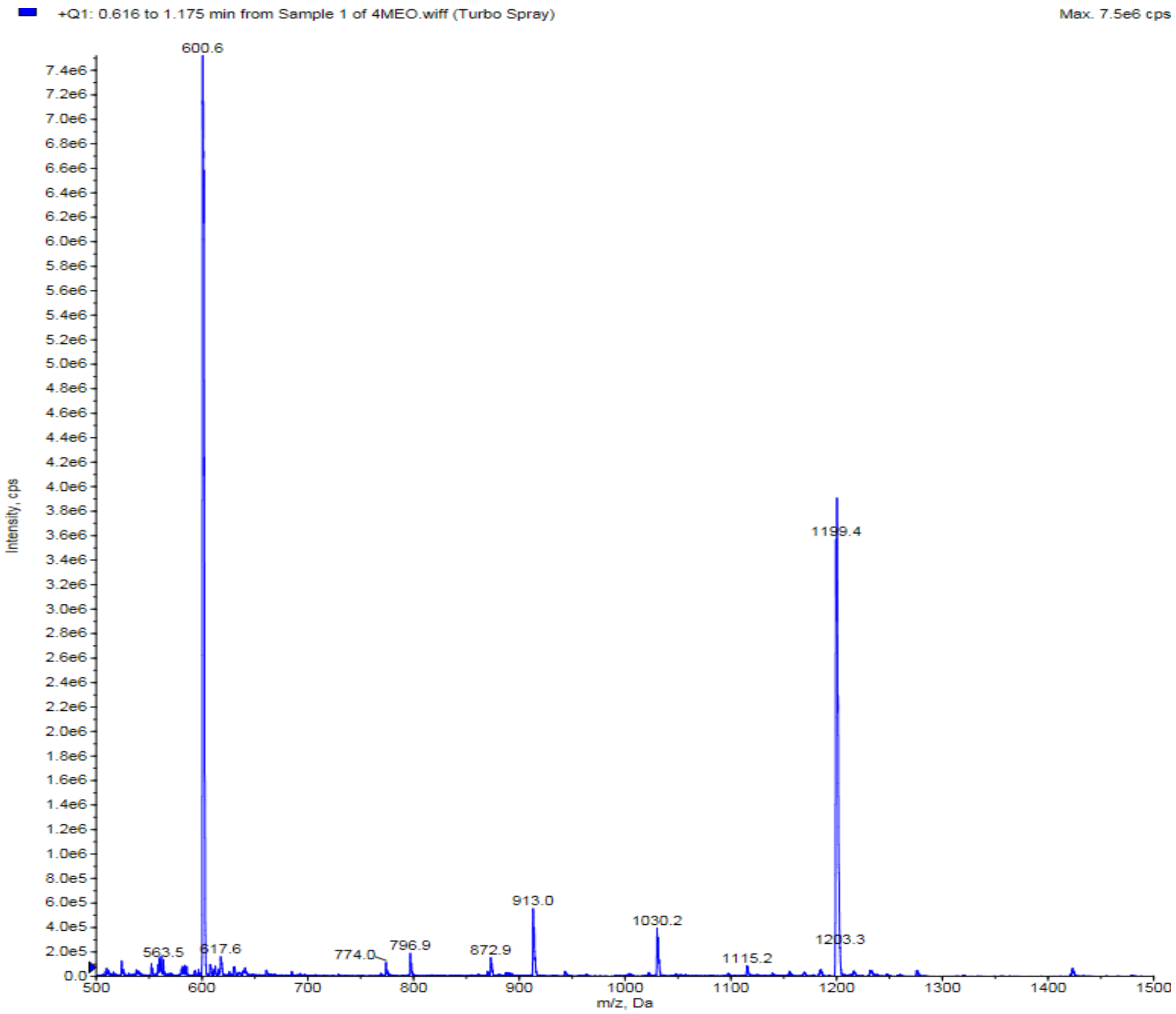

Figure S12. ESI-MS results of N4,N4'-bis(4-(4,5-bis(4-methoxyphenyl)-1-phenyl-1H-imidazol-2-yl)phenyl)-N4,N4'-diphenylbiphenyl-4,4'-diamine (4, TPI-4MEO, M_W=1197.42).

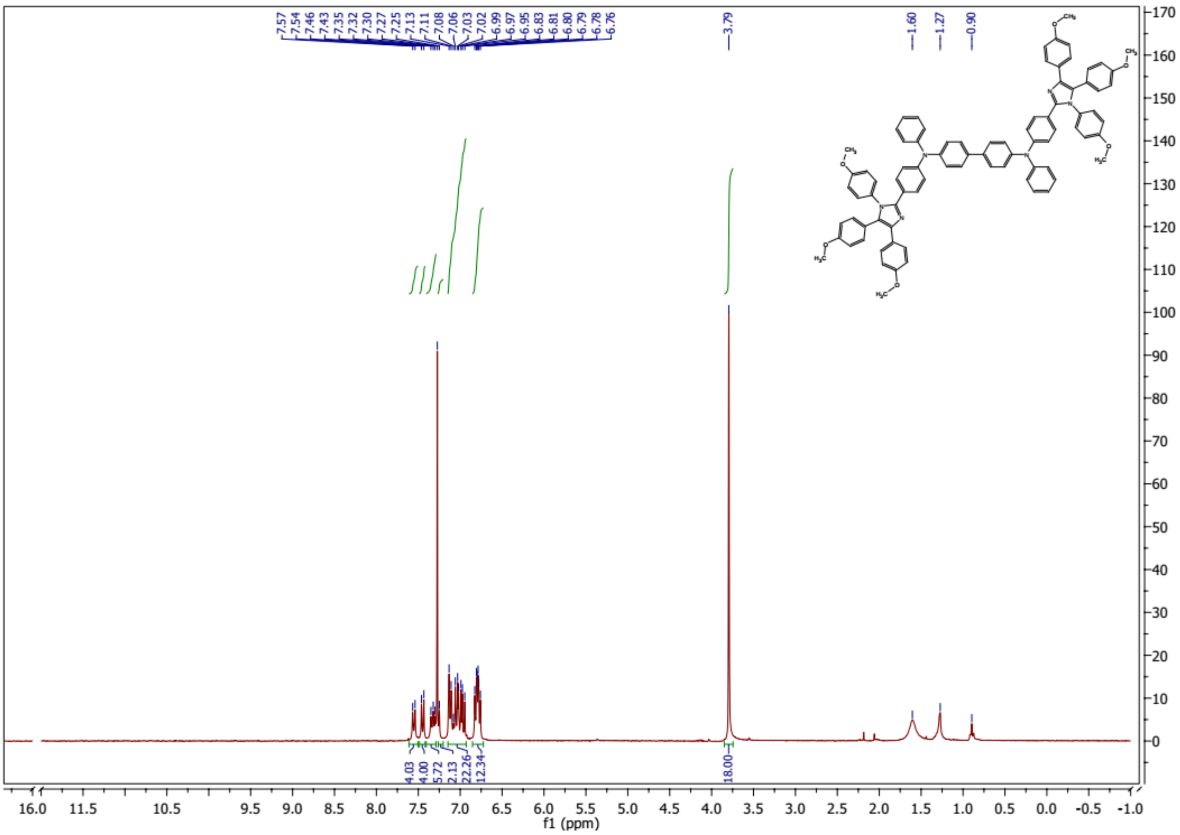

Figure S13. ^{1}H NMR (300 MHz, $CDCl_3$) of N4,N4'-diphenyl-N4,N4'-bis(4-(1,4,5-tris(4-methoxyphenyl)-1H-imidazol-2-yl)phenyl)biphenyl-4,4'-diamine (5, TPI-6MEO).

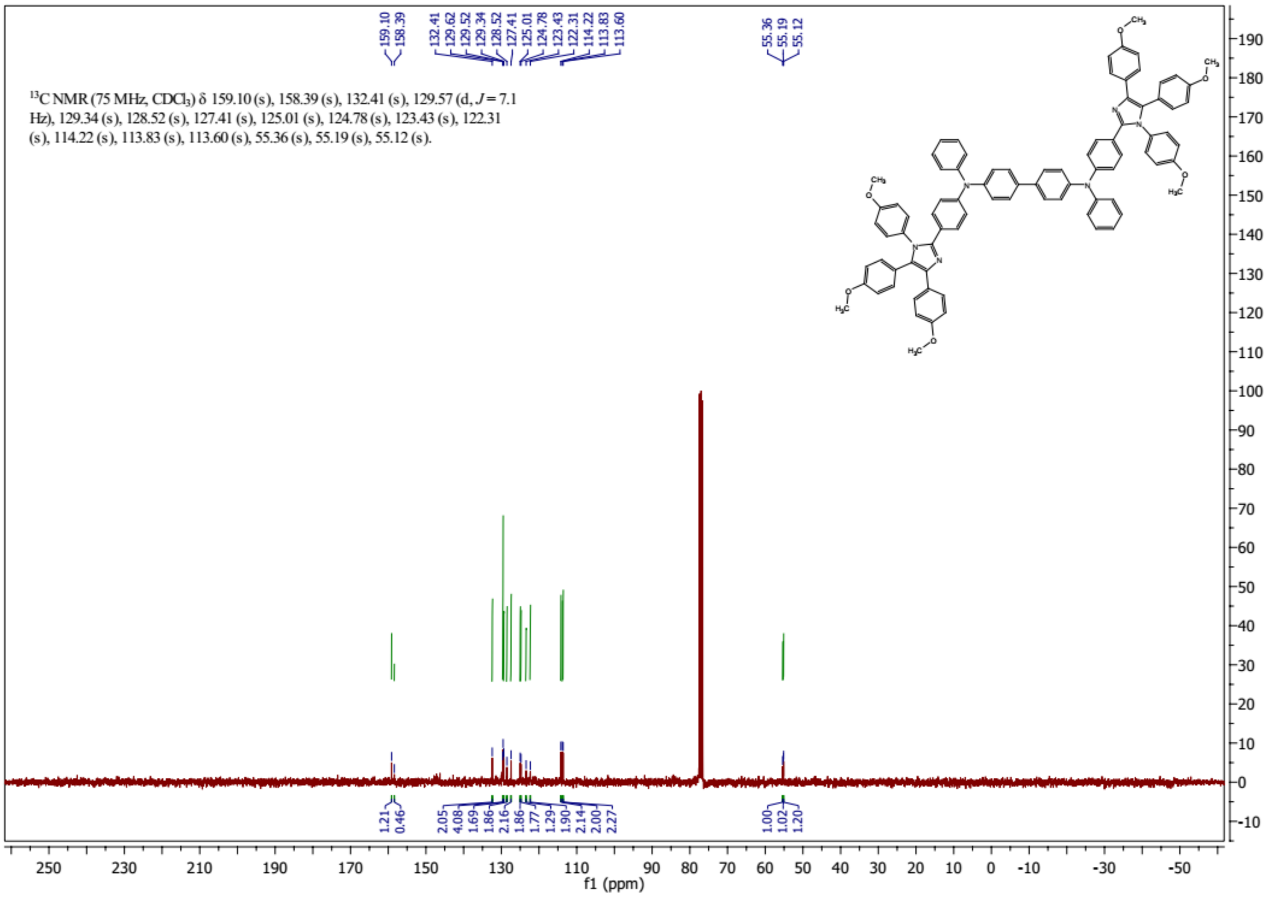

Figure S14. ^{13}C NMR ($CDCl_3$) of N4,N4'-diphenyl-N4,N4'-bis(4-(1,4,5-tris(4-methoxyphenyl)-1H-imidazol-2-yl)phenyl)biphenyl-4,4'-diamine (5, TPI-6MEO).

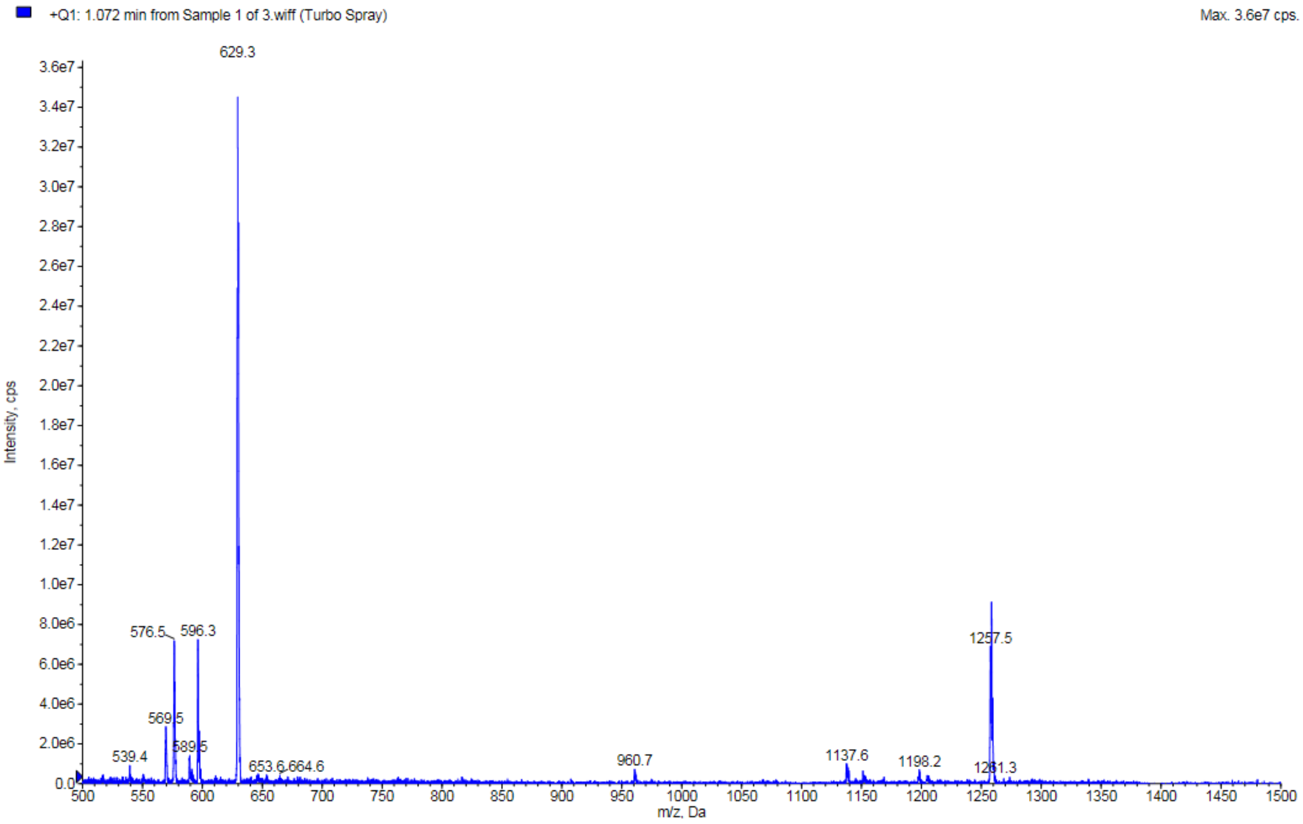

Figure S15. ESI-MS results of N4,N4'-diphenyl-N4,N4'-bis(4-(1,4,5-tris(4-methoxyphenyl)-1H-imidazol-2-yl)phenyl)biphenyl-4,4'-diamine (5, TPI-6MEO, M_W=1257.48).

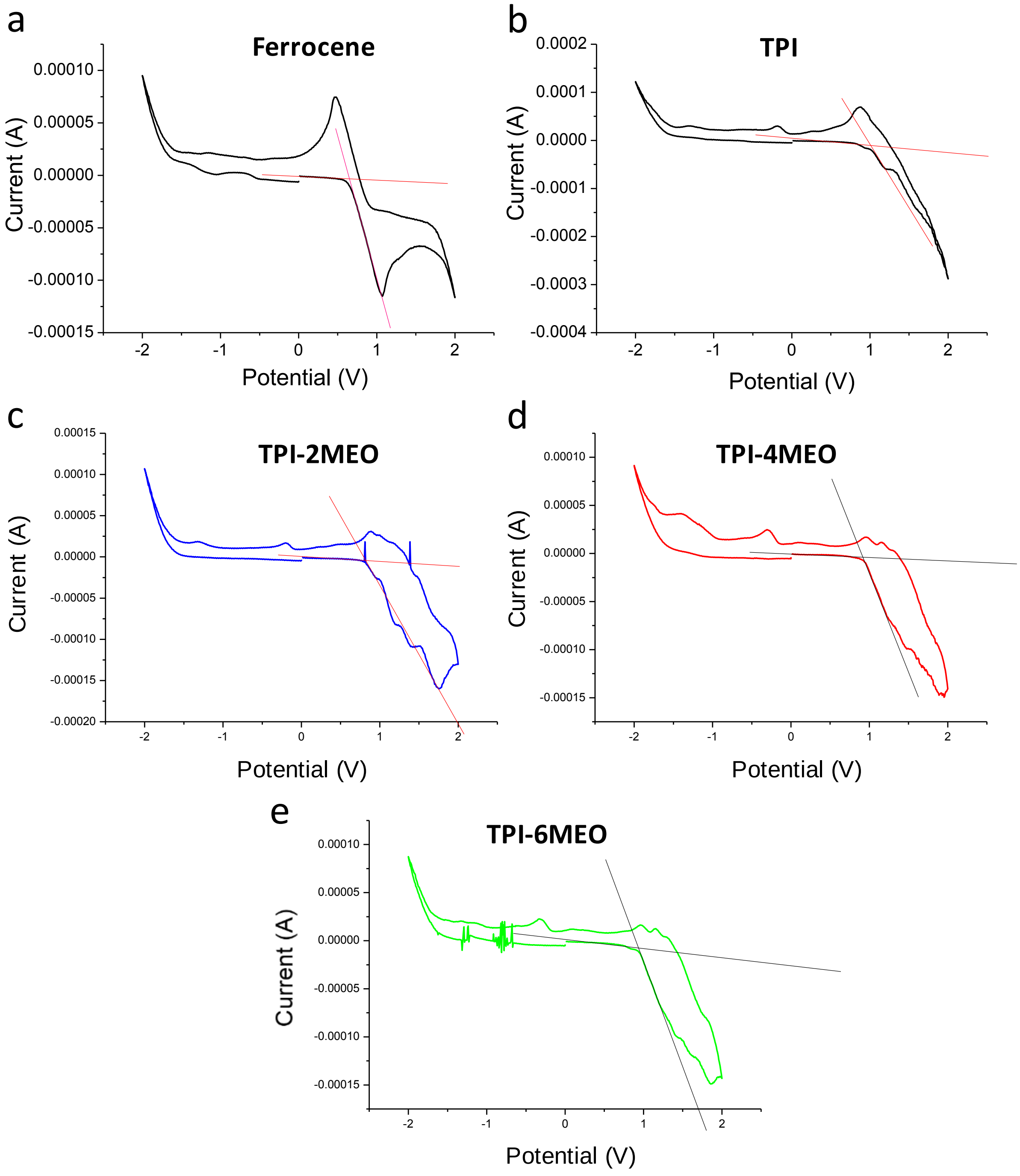

Figure S16. Cyclic voltammetry (CV) results of (a) ferrocene, (b) TPI, (c) TPI-2MEO, (d) TPI-4MEO, and (e) TPI-6MEO.

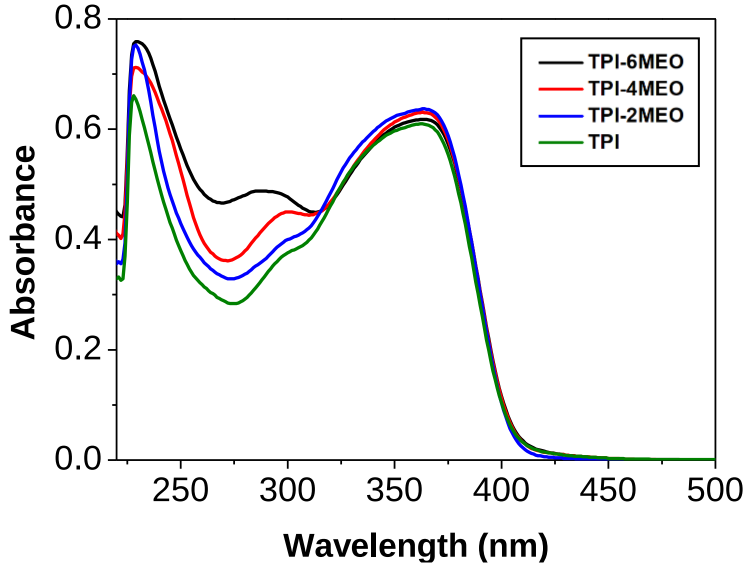

Figure S17. Absorbance of TPI, TPI-2MEO, TPI-4MEO, and TPI-6MEO.

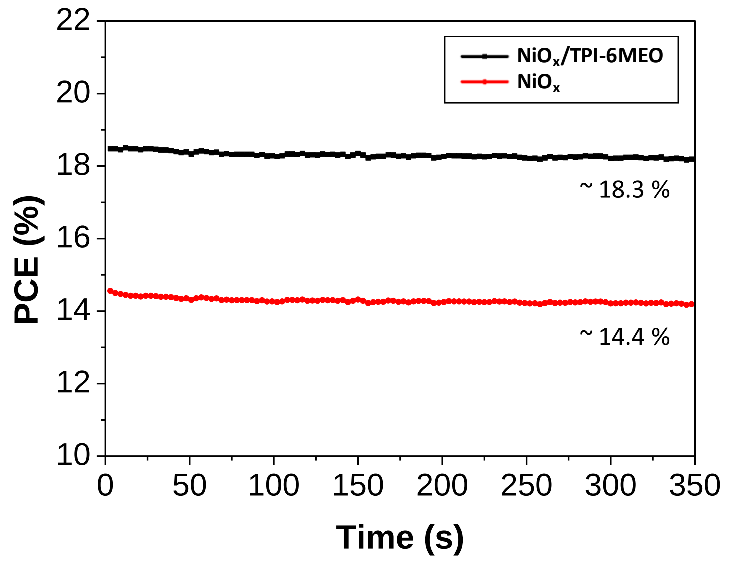

Figure S18. PCEs measured at the maximum power point (MPP) for PSCs with and without TPI-6MEO on NiO_x.

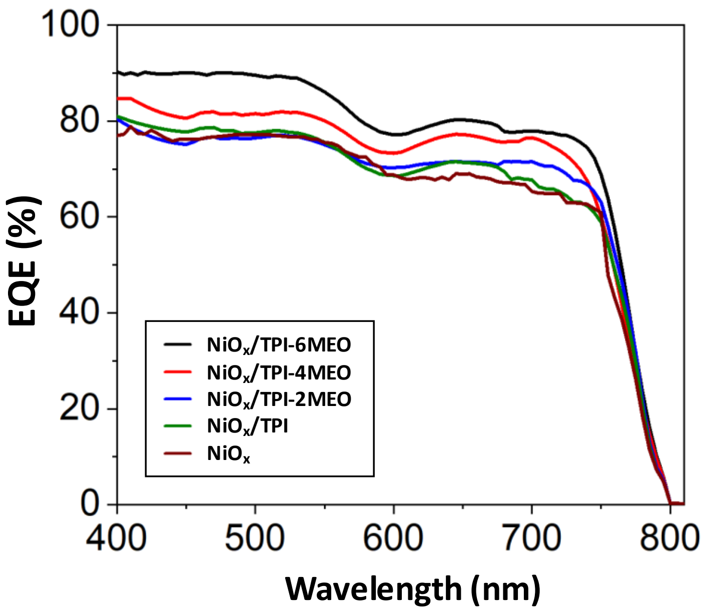

Figure S19. External quantum efficiency (EQE) spectra of PSCs with various hole transport materials.

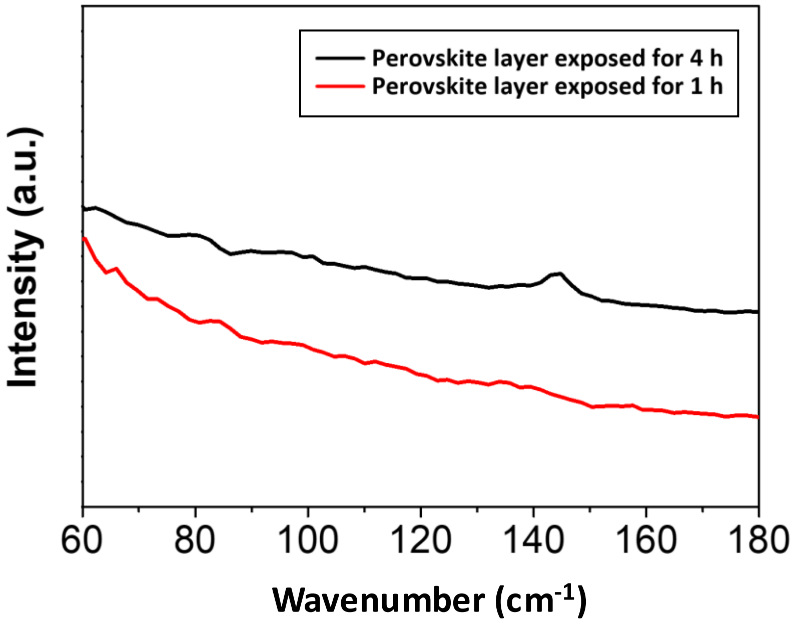

Figure S20. Raman spectra of perovskite-only layer after air exposure.

Table S1 | Fitted parameters of PL decay curves of perovskite on various HTMs, excited by 670 nm laser (the values of the goodness-of-fit parameter (χ^2) are all close to 1.0).

	A_1	τ_1(ns)	A_2	τ_2(ns)	A_3	τ_3(ns)	τ_{avg}(ns)
Perovskite	24.8 %	48.1	44.2 %	12.4	30.9 %	2.1	18.1
NiO_x/perovskite	6.8 %	108.5	55.8%	4.6	37.4 %	21.4	17.9
NiO_x/TPI/perovskite	5.4 %	105.7	61.8 %	3.5	32.9 %	17.4	13.6
NiO_x/TPI-2MEO/perovskite	6.9 %	87.8	63.1 %	2.6	30.0 %	16.4	12.6
NiO_x/TPI-4MEO/perovskite	10.9 %	38.7	45.7 %	1.8	43.4 %	8.7	8.8
NiO_x/TPI-6MEO/perovskite	6.6 %	52.5	23.0 %	8.3	70.4 %	0.9	6.1

where, $Counts(t) = A_1\exp(-t/\tau_1) + A_2\exp(-t/\tau_2) + A_3\exp(-t/\tau_3)$
τ_{avg}: amplitude weighted average lifetime
